# Online Test Strategy Generation Based on Delayed-Feedback Value Learning

**DOI:** 10.3390/s26216997

**Published:** 2026-11-05

**Authors:** Haiquan Cao, Meiqi He, Jingyuan Wang, Xiaowu Chen, Zhen Liu

**Affiliations:** 1 School of Automation Engineering, University of Electronic Science and Technology of China, Chengdu 611731, China; chqstar@126.com (H.C.); 2024060901024@std.uestc.edu.cn (M.H.); jywang@uestc.edu.cn (J.W.); xwchen@uestc.edu.cn (X.C.); 2 State Grid Sichuan Electric Power Company, Chengdu 610041, China

**Keywords:** design for testability, test strategy generation, online fault diagnosis, delayed-feedback value learning

## Abstract

Design for testability configures test points and plans tests during product design to support fault detection and isolation in service. Existing test strategy generation (TSG) methods mainly address offline diagnosis. As demand for uninterrupted operation grows, downtime and economic losses become more significant, shifting maintenance toward online diagnosis. However, offline methods optimize complete diagnostic processes without directly accounting for isolation gains within a limited time. Their direct application can therefore yield a low fault isolation rate (FIR). To address this problem, this paper proposes an online TSG method based on delayed-feedback value learning (DFVL). First, an optimization problem is formulated using a multi-signal flow graph and a finite time constraint. Second, a DFVL framework is developed using value recursion and feasible subtree returns. It comprises Bellman-based features, balanced feasible completions, delayed updates with unweighted normalized residuals, historical-tree retention, model-derived MAP candidates, and bounded local search. Across 90 synthetic models and 12 budgets, mean FIR was 83.53%, compared with 77.96% for Mix1 and 73.24% for DIG. At normalized Time 36, device-model FIR was 75.90%, versus 53.15% for Mix1 and 61.86% for DIG. FIR denotes a model-based correct terminal MAP decision probability under single-fault, deterministic-response, and fixed-duration assumptions.

## 1. Introduction

### 1.1. Research Motivation

Design for testability involves configuring test points and planning tests during product design to generate diagnostic strategies [1,2]. Traditional design for testability mainly addresses offline fault diagnosis [3,4]. Under offline conditions, test strategy generation (TSG) generally does not require strict time limits. Its main objective is to reduce the average test cost by optimizing test selection and sequencing while achieving complete fault isolation [5,6].

However, as the demand for continuous equipment operation and the cost of downtime increase, offline TSG has become less able to meet the requirements of online fault diagnosis. This has driven the development of TSG for online applications. Unlike offline TSG, online TSG must account for explicit time constraints. Its primary objective is no longer complete isolation, but the highest possible fault isolation rate (FIR) within a limited time. Traditional offline methods mainly optimize the complete diagnostic process. They generally do not use the isolation gains obtainable within a limited time as a basis for decision making. Consequently, applying offline methods directly to online problems can yield low fault isolation gains within the available test time.

### 1.2. Literature Review

Existing offline TSG methods can be broadly divided into two categories: greedy algorithms based on local test evaluation functions, and global planning algorithms based on intelligent optimization and combinatorial search.

The basic idea of a greedy algorithm is to select the locally optimal test at each decision node according to the current evaluation values of candidate tests. It does not search the entire strategy space globally, and is therefore fast and straightforward to implement [7,8]. Tian et al. combined the fault detection weight with information entropy and proposed two greedy algorithms, Mix1 and Mix2. These algorithms improve the applicability of a single evaluation metric across different D-matrices [7]. For diagnostic test selection in multimode systems, Krysander et al. used a fault signature matrix and a greedy algorithm to select residual sets progressively. Their method reduces the number of tests while preserving diagnosability in each operating mode [8]. Zhang et al. combine information gain with fault-detection weighting in DIG [9]. Related greedy sensor-selection methods address experimental design [10] and observability-Gramian optimization [11]. Such methods generally select the next test using an evaluation function at the current node or limited look-ahead, and offer high computational efficiency. However, if the evaluation does not account for isolation gains before the deadline, directly truncating the generated strategy may fail to use the diagnostic window effectively.

Global planning algorithms typically use intelligent optimization or combinatorial search to jointly optimize the test set, test sequence, and diagnostic tree structure [12,13,14,15,16,17,18,19]. Yu et al. encoded diagnostic strategies using a fault–test dependency matrix. They used a quantum-inspired genetic algorithm to jointly optimize the expected test cost and the number of tests used [12]. Li et al. developed a data-driven test selection model and used an improved discrete binary particle swarm optimization algorithm to solve test selection under multiple faults [13]. They subsequently described test dependencies using a conditional joint distribution and a deep copula to reduce detection and maintenance costs [14]. Hu et al. developed a test point selection framework based on a fault response matrix to obtain high fault coverage with fewer test points [15]. Zhu et al. established a four-valued dependency matrix for systems with complex multivalued attributes and designed a diagnostic strategy optimization method. This further integrates testability modeling with TSG [16]. Li et al. model dependent test outcomes and multiple-fault ambiguity for test selection [20]. Kulkarni et al. developed a multiobjective sensor selection framework to improve the fault detection rate (FDR) while reducing the total sensor cost [17]. Wang et al. further formulated a test selection optimization model with FDR and FIR constraints for multivariable and multifault scenarios [18]. Li et al. developed a sequential fault diagnosis strategy for high-speed train traction systems with unreliable tests [19]. These sensor-selection and unreliable-test studies consider FDR/FIR requirements or response uncertainty, but their objectives differ from maximizing the terminal MAP return under a separate deadline on every diagnostic path. Ash and Hayes-Roth associate diagnostic-tree nodes with actions that remain available when a deadline is reached [21]. Bayer-Zubek formulates test selection and stopping as a Markov decision process and uses AO* search to minimize expected test and misdiagnosis costs [22]. Liang et al. apply Q-learning to diagnostic policies with imperfect tests [23]. Yang et al. use a deep Q-network for analog-circuit test-point selection under a cost constraint [24]. Zhang et al. apply deep reinforcement learning to multi-valued dependency matrices to reduce the steps or expected cost of complete isolation [25]. These methods improve the adaptability of test selection and TSG under complex conditions.

Although the above TSG methods have been widely applied, they do not share a single objective. Complete-process cost optimization and finite-window MAP return need not select the same test sequence. The present work plans a separate budget-feasible strategy for each given binary D/P/C model and time limit, using achieved feasible subtree returns as feedback.

### 1.3. Research Content and Contributions

To address these problems, this paper proposes an online TSG method based on delayed-feedback value learning. First, an online TSG optimization problem is formulated by combining a multi-signal flow graph model with a finite time constraint. The basic FIR monotonicity identity supports feasible return bounds. Further, a delayed-feedback value learning (DFVL) framework is developed by combining value recursion with feasible subtree feedback. The framework constructs features from Bellman recursion, generates training samples through subtree completion and return records, and updates the network using a delayed-feedback equation with whole-tree backup. Historical feasible trees, model-derived MAP candidates, state-matched subtree reuse, and bounded local search are integrated into the final strategy construction. The main contributions are as follows:

1. A TSG optimization problem is formulated for online fault diagnosis with limited test time, explicitly defining the objective of maximizing FIR within that time.
2. Bounded action-value features and delayed feedback from completed feasible subtrees provide normalized learning targets, using balanced completions and unweighted residuals.
3. The learned values are combined with historical feasible-tree retention, model-derived candidates and bounded search to construct a feasible diagnostic strategy for each known model and budget.

The remainder of this paper is organized as follows. Section 2 formulates the online TSG optimization problem and checks its motivating approximation. Section 3 states the return identity used by feasible bounds. Section 4 presents the proposed DFVL framework. Section 5 reports the evaluations, and Section 6 concludes the paper.

## 2. MSFG Model and Formulation of the Online TSG Problem

### 2.1. MSFG

The model assumes that exactly one of the listed faults is active during a diagnosis. The priors describe these mutually exclusive fault hypotheses; a healthy-state detection problem and simultaneous multiple faults are not evaluated.

The multi-signal flow graph (MSFG) model describes system fault states, available tests, and their relationships. It provides a basis for TSG and diagnostic decision tree construction [26,27], and is usually represented by the tuple {S,P,T,C,D} [28,29]:

1. The candidate fault set is $S=\{s_{1},s_{2},\ldots ,s_{m}\}$, where $s_{i}$ denotes the *i*th system fault state and *m* is the total number of candidate fault states.
2. The prior fault probability vector is $P={\left(p_{1},p_{2},\ldots ,p_{m}\right)}^{T}$. The designer determines $p_{i}$ from system information and failure mode, effects, and criticality analysis (FMECA). It represents the prior probability that the system is in state $s_{i}$, conditional on a fault having occurred, and satisfies $p_{i}\geq 0$ and $\sum_{i=1}^{m}p_{i}=1$.
3. The available test set is $T=\{t_{1},t_{2},\ldots ,t_{n}\}$. Here, $t_{j}$ denotes the *j*th test that can be performed on the system, and *n* is the total number of available tests.
4. The test time vector is $C={\left(c_{1},c_{2},\ldots ,c_{n}\right)}^{T}$. The designer specifies $c_{j}$ according to the actual measurement conditions. It represents the time required to perform test $t_{j}$ and satisfies $c_{j}>0$.
5. The fault–test dependency matrix is $D={\left(d_{ij}\right)}_{m\times n}$, where $d_{ij}\in \left\{0,1\right\}$. If test $t_{j}$ responds to fault $s_{i}$, then $d_{ij}=1$; otherwise, $d_{ij}=0$.

### 2.2. Formulation of the Online TSG Optimization Problem

At a node with candidate fault set A⊆S, the maximum a posteriori (MAP) rule is the optimal diagnostic decision rule when misclassification costs are equal [30]. The candidate fault with the highest probability in *A* should be selected as the diagnostic result. In this paper, FIR is measured by the probability of a correct MAP decision. The conditional probability of a correct decision at this node is $\max_{s_{i}\in A}p_{i}/\sum_{s_{i}\in A}p_{i}$, and its contribution to the overall FIR is

(1) $$\mathrm{FIR}\left(A\right)=\max_{s_{i}\in A}p_{i}.$$

Throughout this paper, FIR denotes the prior-weighted probability that the terminal MAP decision is correct under the supplied model. A leaf containing several candidate faults contributes the prior of its MAP representative. This criterion does not require every terminal candidate set to be a singleton.

Offline TSG generally minimizes the average test cost subject to complete fault isolation. By contrast, online tests must be completed within an engineering-defined diagnostic window, and their objective is to maximize FIR within a limited time. Let π denote a diagnostic decision tree, P(π) the set of all root-to-leaf paths, and L(π) the collection of candidate fault sets at its leaves. Let Time be the maximum allowable diagnostic time. Because the candidate fault sets at different leaves are disjoint, the overall FIR equals the sum of their contributions. Therefore, the online TSG optimization problem can be expressed as

(2) $$\begin{array}{cc} \max_{\pi } & \mathrm{FIR}\left(\pi \right)=\sum_{A\in L(\pi )}\mathrm{FIR}\left(A\right),\\ s.t. & \sum_{t_{j}\in \xi }c_{j}\leq \mathrm{Time},\;\forall \;\xi \in P\left(\pi \right). \end{array}$$

**Figure 1:**
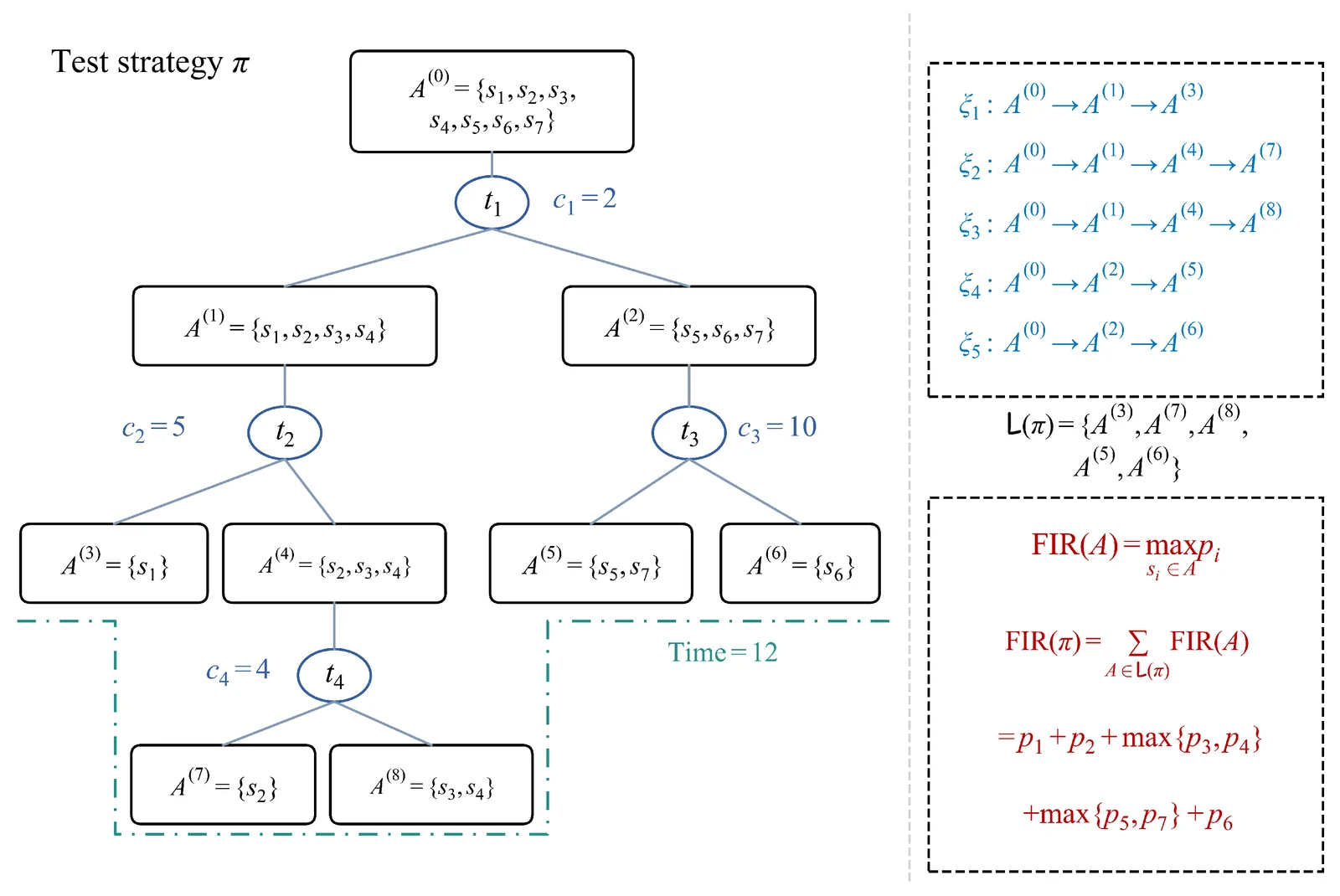
Diagnostic tree under a time constraint and an example FIR calculation.

A calculation example is shown in Figure 1. The dashed line indicates the test deadline. Tests that have not been completed before this deadline are excluded from the fault decision at the leaf node.

### 2.3. Loss in the Return of Complete Diagnostic Strategies Under Time Limits

The supplied priors are inputs to strategy generation. For the controlled simulations, three fault grades use relative weights 100:10:1, which are normalized to obtain *P*. This mapping is a modeling choice, rather than a numerical ratio prescribed by MIL-STD-1629A, whose probability classes provide a qualitative classification [31]. The engineering case uses the supplied grade assignments; an alternative uniform prior is evaluated separately.

Let the numbers of faults in the three levels be $m=(m_{1},m_{2},m_{3})$, with weights $w_{1}>w_{2}>w_{3}$, and let q∈{1,2,3} index the prior probability levels. Tests are divided into $L_{C}$ cost levels. The cost and number of tests at level *ℓ* are $c_{\ell },n_{\ell }$, respectively, with $n=(n_{1},\ldots ,n_{L_{C}})$ and $n=\sum_{\ell =1}^{L_{C}}n_{\ell }$. Define $Z=\sum_{q}w_{q}m_{q}$ and $W\left(r\right)=\sum_{q}w_{q}r_{q}$. Let M(r) be the largest fault weight in composition r, with a value of 0 for an empty node. Each fault has prior probability $w_{q}/Z$, and the same *Z* is used at every child node. The true loss is defined as the difference between the optimal online FIR and the FIR of a truncated entropy tree under the same deadline. The expectation is taken over random dependency matrices whose entries independently follow Bernoulli(ρ), and over uniform selection when entropy values are tied. Let $\pi_{\mathrm{ent}}^{\left(t\right)}$ denote the feasible strategy obtained by truncating a complete entropy tree at budget *t*. Let $\hat{G}\left(t\right)$ estimate the unnormalized mean return of the truncated entropy tree, and let $\hat{U}\left(m,n,t\right)$ estimate the optimal unnormalized return of online planning. Then,

(3) $$\begin{array}{cc} \bar{\varepsilon }\left(t\right) & =E\;\left[\max_{\begin{array}{c} \pi \;\mathrm{satisfies}\;\mathrm{Equation}\;(2)\\ \mathrm{with}\;\mathrm{budget}\;t \end{array}}\mathrm{FIR}\left(\pi \right)-\mathrm{FIR}\left(\pi_{\mathrm{ent}}^{\left(t\right)}\right)\right]\geq 0,\\ \bar{\varepsilon }\left(t\right) & \approx \tilde{\varepsilon }\left(t\right)=\frac{\hat{U}\left(m,n,t\right)-\hat{G}\left(t\right)}{Z}. \end{array}$$

The truncated strategy is itself feasible, so the true difference is nonnegative. The analytical estimate assumes node independence, independence of the remaining columns, and closure based on the conditional means of successor returns. The derivation is given below.

For a node with composition r, $r_{q}$ is the number of faults at prior level *q*. Let k denote the composition of faults with test response 1, where $k_{q}$ is the number of such faults at that level. The composition for response 0 is r−k. Define $K_{r}=\prod_{q}\left\{0,\ldots ,r_{q}\right\}$ and the outcome entropy as $h_{r}\left(k\right)=H_{2}\left[W\left(k\right)/W\left(r\right)\right]$. Let $F_{r}$ be the distribution function of this entropy. Among $N_{\mathrm{cand}}$ candidate tests, the probability that the maximum-entropy test has response-1 composition k is

(4) pr(k)=∏q=13rqkqρ∑qkq(1−ρ)∑q(rq−kq),ωr,Ncand(k)=pr(k)Fr(h)−Fr(h−)Fr(h)Ncand−Fr(h−)Ncand,h=hr(k).

Here, $H_{2}$ is the binary entropy, and $F_{r}\left(h^{-}\right)$ is the probability of an entropy value strictly below *h*. Tests with tied entropy values are selected with equal probability. Define $K_{r}^{+}=K_{r}\setminus \left\{0,r\right\}$. Let $\alpha_{d}\left(r\right)$ be the mean number of nodes with this composition at depth *d* in the complete entropy tree. The initial values are $\alpha_{0}\left(m\right)=1$ and 0 for all other compositions. Let $r^{\prime }$ be the composition to be counted at the next depth, and let $\gamma_{d+1}$ be the mean increment in the unnormalized MAP return at test d+1. Dividing this increment by *Z* gives the FIR increment. The indicator $1_{\left\{\cdot \right\}}$ equals 1 when its condition holds and 0 otherwise. From the binary tree structure and the additivity of MAP returns,

(5) $$\begin{array}{cc} \alpha_{d+1}\left(r^{\prime }\right) & =\sum_{r}\alpha_{d}\left(r\right)\sum_{k\in K_{r}^{+}}\omega_{r,n-d}\left(k\right)\left[1_{\left\{r^{\prime }=k\right\}}+1_{\left\{r^{\prime }=r-k\right\}}\right],\\ \gamma_{d+1} & =\sum_{r}\alpha_{d}\left(r\right)\sum_{k\in K_{r}^{+}}\omega_{r,n-d}\left(k\right)\left[M\left(k\right)+M\left(r-k\right)-M\left(r\right)\right]. \end{array}$$

The sums include only nodes that can be split further, and the maximum depth is $d_{\max}=\min\left\{n,\sum_{q}m_{q}-1\right\}$. Because the entropy criterion does not use costs, test costs along a path are sampled without replacement from a finite population. Let $z_{\ell }$ be the number of tests drawn from cost level *ℓ* among the first *d* tests. The probability that these *d* tests are completed within Time and the estimated mean return of the truncated entropy tree are

(6) Bd(Time)=nd−1∑0≤zℓ≤nℓ,∑ℓzℓ=d∑ℓcℓzℓ≤Time∏ℓ=1LCnℓzℓ,G^(Time)=M(m)+∑d=1dmaxγdBd(Time).

Online planning, in contrast, considers both the test-induced partition and the remaining time. Let $u=(u_{1},\ldots ,u_{L_{C}})$ denote the remaining numbers of tests at each cost level. Let $\hat{U}\left(r,u,t\right)$ estimate the optimal return with remaining time *t*, and define $A=\{\ell :u_{\ell }>0,c_{\ell }\leq t\}$. For a candidate test at cost level *ℓ*, the successor return is $R_{\ell }\left(k\right)$; when no effective partition is obtained, it is set to M(r). Define $A_{\ell }\left(v\right)=\sum_{k}p_{r}\left(k\right)1_{\left\{R_{\ell }\left(k\right)\leq v\right\}}$. Sort M(r) and all distinct candidate return values as $v_{0}=M\left(r\right)<\cdots <v_{J}$, giving J+1 distinct values. The mean of the discrete maximum then gives the recursion

(7) $$\begin{array}{cc} R_{\ell }\left(k\right) & =\hat{U}\left(k,u-e_{\ell },t-c_{\ell }\right)+\hat{U}\left(r-k,u-e_{\ell },t-c_{\ell }\right),k\in K_{r}^{+},\\ \hat{U}\left(r,u,t\right) & =v_{0}+\sum_{j=0}^{J-1}\left(v_{j+1}-v_{j}\right)\left[1-\prod_{\ell \in A}A_{\ell }{\left(v_{j}\right)}^{u_{\ell }}\right]. \end{array}$$

Here, $e_{\ell }$ is a unit vector. The return is 0 for an empty node, and M(r) when only one fault remains or no feasible test is available. Under the approximations of node independence, independence of the remaining columns, and conditional-mean closure of successor returns, this recursion estimates the online return in the random-matrix model.

For example, consider four faults with prior probabilities (100,10,1,1)/112. A test of duration 1 partitions the faults into $\left\{s_{1},s_{3}\right\}$ and $\left\{s_{2},s_{4}\right\}$. A test of duration 3 partitions them into $\left\{s_{1}\right\}$ and $\left\{s_{2},s_{3},s_{4}\right\}$. The latter has an outcome entropy of approximately 0.49 bits, exceeding the former’s 0.46 bits. Thus, a pure entropy criterion selects the duration-3 test first. With a budget of 1, that test remains incomplete, and FIR stays at 100/112. Performing the duration-1 test first instead gives 110/112. This example shows that directly truncating a strategy generated by an entropy criterion for the complete process can be suboptimal.

The approximation was checked on 30 independent 8×8 matrices with ρ=0.50, fault-grade counts (1,2,5), weights (100,10,1), test-cost counts (1,2,5) for costs (1,3,10), and the fixed absolute budget Time=6. Complete exact dynamic programming supplied the optimum for each matrix; the entropy-tree comparison used uniform selection among tied entropy values. The approximate optimal and entropy-tree mean FIR values were 94.86% and 83.93%, compared with Monte Carlo means of 93.41% and 83.01%. Their 95% *t* intervals across 30 independent models (29 degrees of freedom) were [91.87,94.96]% and [81.13,84.90]%, respectively. The estimated loss was 10.93 percentage points, versus an observed mean of 10.40 points, with an interval of [7.97,12.83]. The approximation-minus-Monte-Carlo discrepancies were therefore 1.45, 0.92 and 0.53 points. This check quantifies error for this setting and does not establish accuracy at larger sizes.

## 3. Mathematical Properties of the Online TSG Problem

### Monotonicity of the Fault Isolation Rate

Feasible diagnostic-tree expansion cannot reduce terminal MAP return. The identity below supports the lower bounds and stopping rules used by DFVL.

First, consider how the objective changes as a diagnostic tree expands. For any leaf with candidate set *A*, suppose a feasible test $t_{j}$ splits it into $A_{j}^{0}$ and $A_{j}^{1}$, corresponding to responses 0 and 1. Equation (1) gives

(8) $$\begin{array}{cc} \Delta (A,j) & =\mathrm{FIR}\left(A_{j}^{0}\right)+\mathrm{FIR}\left(A_{j}^{1}\right)-\mathrm{FIR}\left(A\right)\\ & =\min\{\mathrm{FIR}\left(A_{j}^{0}\right),\mathrm{FIR}\left(A_{j}^{1}\right)\}\geq 0. \end{array}$$

The fault with the highest probability in *A* must belong to one of the child nodes. That child therefore retains the original maximum, while the other child adds a nonnegative isolation gain. Since the tree FIR is the sum of its leaf contributions, expanding any leaf without violating the time constraint makes the whole-tree FIR monotonically nondecreasing.

Each diagnostic path can be expanded using feasible tests until no effective test can be completed within the remaining time, or the candidate faults have been completely isolated. If different faults have identical response signatures across all remaining tests, the node cannot obtain any further isolation gain and should also terminate.

## 4. An Online TSG Method Based on Delayed-Feedback Value Learning

Online TSG can be formulated as a time-constrained finite-horizon decision process. The state *x* consists of the current ambiguous fault set, the remaining tests, and the remaining time; an executable test $t_{j}$ is an action; and the reward is the probability that the terminal MAP decision is correct. The ideal heuristic evaluation function (HEF) is the optimal long-term value $Q^{*}\left(x,j\right)$ of a state–test pair within the remaining time and can be evaluated recursively using the Bellman optimality principle.

**Figure 2:**
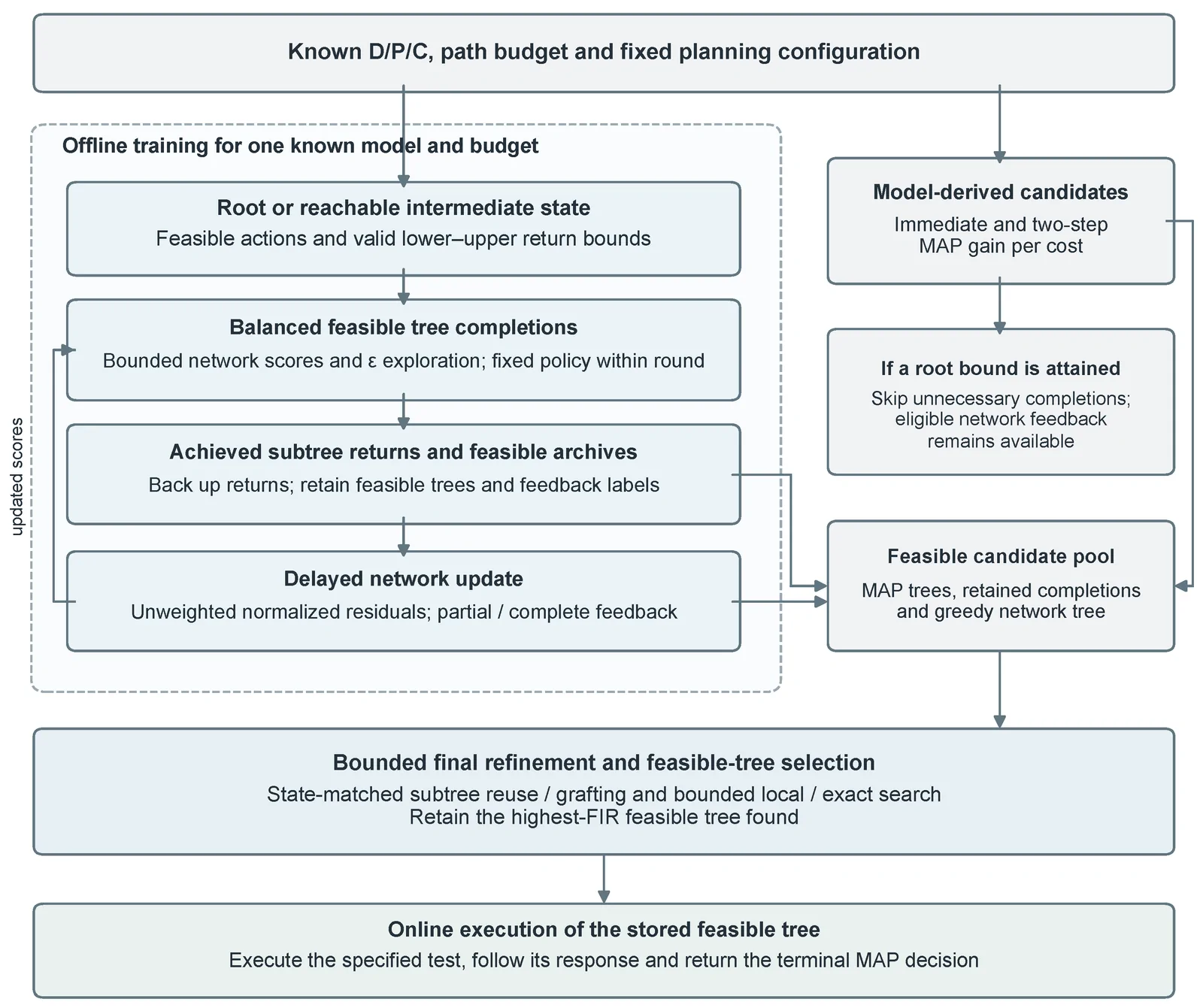
DFVL planning for a known model and budget, followed by online traversal of the stored feasible tree.

DFVL learns bounded action values from the returns of completed feasible subtrees. Those returns update a strategy archive and provide delayed labels for multiple state–test pairs. The complete planning procedure also includes model-derived MAP candidates, state-matched subtree reuse, and bounded local search. Figure 2 summarizes the procedure.

### 4.1. Construction of Action-Value Features

The inputs to the action-value network must first be specified. Let the current node state be x=(A,U,b), where *A* is the ambiguous fault set, *U* is the set of available tests that have not yet been performed, and *b* is the remaining time. Let $w\left(A\right)=\sum_{s_{i}\in A}p_{i}$ denote the probability mass of this candidate set. Its FIR contribution is defined by Equation (1). The index set of currently feasible tests is $J\left(x\right)=\{j:t_{j}\in U,\;w\left(A\right)>0,\;c_{j}\leq b,\;A_{j}^{0}\neq ⌀,\;A_{j}^{1}\neq ⌀\}$. For a candidate first test $t_{j}$, let T(x,j) be the set of all feasible binary subtrees that start with $t_{j}$ and whose remaining duration on every path does not exceed *b*. The ideal HEF is then

(9) $$Q^{*}\left(x,j\right)=\max_{\pi \in T(x,j)}\frac{\sum_{A^{\prime }\in L\left(\pi \right)}\mathrm{FIR}\left(A^{\prime }\right)}{w(A)},\;x_{j}^{y}=\left(A_{j}^{y},U\setminus \left\{t_{j}\right\},b-c_{j}\right),$$

where $A_{j}^{y}=\left\{s_{i}\in A:d_{ij}=y\right\}$ and y∈{0,1}. Let $V^{*}\left(x\right)$ denote the optimal conditional FIR at state *x* when immediate stopping is allowed. The Bellman optimality principle gives

(10) $$\begin{array}{cc} Q^{*}\left(x,j\right) & =\sum_{\begin{array}{c} y=0,1\\ w(A_{j}^{y})>0 \end{array}}\frac{w(A_{j}^{y})}{w(A)}V^{*}\left(x_{j}^{y}\right),\\ V^{*}\left(x\right) & =\left\{\begin{array}{cc} \mathrm{FIR}(A)/w(A), & J(x)=⌀,\\ \max\{\mathrm{FIR}\left(A\right)/w\left(A\right),\max_{j\in J(x)}Q^{*}\left(x,j\right)\}, & J(x)\neq ⌀, \end{array}\right.\\ V^{*}\left(\left(S,T,\mathrm{Time}\right)\right) & =\max_{\pi \;\mathrm{satisfying}\;\mathrm{Equation}\;(2)}\mathrm{FIR}\left(\pi \right). \end{array}$$

The root probability mass is w(S)=1, so the local conditional value agrees with the overall objective in Equation (2). Branches with zero probability mass are excluded from the weighted sum. Equation (10) decomposes the long-term value of a test into the gain from the current partition and the future values of the two child states. The latter are jointly constrained by the remaining time and fault separability. Accordingly, the input features are extracted from three aspects: immediate gain, remaining time and feasible testing depth, and future separability.

**(1)**  **Immediate gain.** The size of the current ambiguous fault set determines the scope for improvement. After a test divides the set into two subsets, the MAP returns of the two child nodes constitute the achieved FIR. The immediate gain Δ(A,j) relative to the value before testing is calculated using Equation (8).
**(2)**  **Remaining time and feasible testing depth.** The current remaining time *b*, the duration $c_{j}$ of the candidate test, and the tests that remain executable at the child nodes jointly determine the depth of subsequent expansion. For any child state $x^{\prime }=\left(A^{\prime },U^{\prime },b^{\prime }\right)$, arrange the durations of all remaining tests in $U^{\prime }$ in ascending order as $c_{\left(1\right)},c_{\left(2\right)},\ldots$. The remaining time imposes the following upper bound on the number of tests along a path:
  (11) $$d\left(x^{\prime }\right)=\min\;\left\{|A^{\prime }|-1,\;\max\left\{r\in \left\{0,\ldots ,\right|U^{\prime }\left|\right\}:\sum_{\ell =1}^{r}c_{\left(\ell \right)}\leq b^{\prime }\right\}\right\}.$$
**(3)**  **Future separability.** Even when sufficient time is available, faults with identical responses cannot be distinguished using the available tests. Faults with the same response vector over $U^{\prime }$ belong to the same signature class. Suppose there are $k(x^{\prime })$ such classes, and let $a_{\left(1\right)}\left(x^{\prime }\right),\ldots ,a_{\left(k\right)}\left(x^{\prime }\right)$ be their maximum prior probabilities in descending order. A binary subtree with depth no greater than $d(x^{\prime })$ can have at most $2^{d(x^{\prime })}$ leaves, and faults within the same signature class cannot be separated further. An upper bound on the subtree FIR is therefore
  (12) $$F\left(x^{\prime }\right)=\sum_{r=1}^{\min\{k\left(x^{\prime }\right),2^{d(x^{\prime })}\}}a_{\left(r\right)}\left(x^{\prime }\right).$$
  With the first test $t_{j}$ fixed, a one-step look-ahead at each of the two child nodes provides a feasible lower bound. Summing their bounds from Equation (12) gives an upper bound:
  (13) $$\begin{array}{cc} \ell_{j}\left(x\right) & =\frac{1}{w(A)}\sum_{y=0}^{1}\left[\mathrm{FIR}\left(A_{j}^{y}\right)+\max_{k\in J(x_{j}^{y})}\Delta \left(A_{j}^{y},k\right)\right],\\ u_{j}\left(x\right) & =\frac{F\left(x_{j}^{0}\right)+F\left(x_{j}^{1}\right)}{w(A)},\;\ell_{j}\left(x\right)\leq Q^{*}\left(x,j\right)\leq u_{j}\left(x\right). \end{array}$$
  If a child node has no feasible test, the corresponding maximum immediate gain in this expression is set to 0.

The information from these three aspects is normalized and supplied to the network. For $x^{\prime }=\left(A^{\prime },U^{\prime },b^{\prime }\right)$, define the five-dimensional child-node feature vector as

(14) $$\psi \left(x^{\prime };x\right)=\left(\frac{w(A^{\prime })}{w(A)},\;\frac{\mathrm{FIR}(A^{\prime })}{w(A^{\prime })},\;\frac{\left|J(x^{\prime })\right|}{\max\{1,|U^{\prime }|\}},\;\frac{d(x^{\prime })}{\max\{1,|A^{\prime }|-1\}},\;\frac{k(x^{\prime })}{|A^{\prime }|}\right).$$

These five quantities represent, respectively, the child-branch probability mass, the current MAP return, the proportion of feasible subsequent tests, the remaining feasible testing depth, and the proportion of distinguishable fault classes. The two child branches are ordered by increasing probability mass. When their masses are equal, they are ordered by response value, 0 before 1. The resulting vectors are denoted by $\psi^{\left(1\right)}$ and $\psi^{\left(2\right)}$. Combining them with the parent-node size and remaining time, the current test duration and immediate gain, and the value bounds gives

(15) $$\begin{array}{cc} \phi (x,j)=( & \left|A\right|/|S|,\;b/\mathrm{Time},\;c_{j}/b,\;\Delta \left(A,j\right)/w\left(A\right),\;\ell_{j}\left(x\right),\;u_{j}\left(x\right),\\ & \psi^{\left(1\right)},\;\psi^{\left(2\right)})\in R^{16}. \end{array}$$

The vector contains 16 normalized features. If Time=0 or no feasible test is available, the procedure terminates without computing action features. The ratio $c_{j}/b$ is computed only when b>0 and $t_{j}$ is executable. Conditional values and their features are defined for states with positive probability mass. A branch with zero probability mass terminates immediately, contributes 0 to the FIR, and has its conditional features set to a zero vector.

### 4.2. Training Sample Generation and Archive Retention

Training samples are generated through state sampling, tree completion, and return recording. Their coverage is controlled by the starting states, exploratory tree completions, and fixed per-state completion quotas.

**(1)**  **State coverage and exploration.** Starting states alternate between the root and a reachable intermediate state of the same supplied model. Intermediate states use feasible prefixes whose target depths cycle from one to six; tests are drawn uniformly and branches according to their prior mass. Each training round collects fresh initial completions for feasible first tests, stopping early if a root tree attains a valid upper bound. Network parameters and exploration probability remain fixed within that round. Each round generates fresh feasible completions, while the feasible-tree archives and feedback labels persist across rounds. With probability ε, a completion selects uniformly among feasible tests other than its current highest-scoring test, when alternatives exist; otherwise it uses that highest-scoring test.
**(2)**  **Tree feedback and the archive.** Let π denote the feasible subtree obtained from each completion; it satisfies the time constraint on every path. Let *v* denote a tree node, $v_{y}$ its child for response *y*, and $A_{v}$ its candidate fault set. For nodes with positive probability mass, the achieved return is backed up from the leaves to the root:
  (16) $$Y_{v}=\left\{\begin{array}{cc} \mathrm{FIR}\left(A_{v}\right)/w\left(A_{v}\right), & v\;\mathrm{is}\;a\;\mathrm{leaf}\;\mathrm{node},\\ \sum_{\begin{array}{c} y=0,1\\ w(A_{v_{y}})>0 \end{array}}\frac{w(A_{v_{y}})}{w(A_{v})}Y_{v_{y}}, & v\;\mathrm{is}\;\mathrm{an}\;\mathrm{internal}\;\mathrm{node}. \end{array}\right.$$
  If a completion starts from x=(A,U,b), then $Y_{\mathrm{root}(\pi )}=\mathrm{FIR}\left(\pi \right)/w\left(A\right)$. For a completion from the root state, this value is the overall FIR of the tree. Branches with zero probability mass contribute zero and are excluded from the conditional-value backup. For each state–first-test pair, the highest achieved conditional value is retained as the label B(x,j). Separately, the state archive retains a highest-return feasible completed subtree for each state. The root archived tree is a final candidate independently of the current greedy network tree. The best achieved value at a state is
  (17) $$B\left(x,j\right)\leftarrow \max\;\left\{B\left(x,j\right),\;Y_{\mathrm{root}(\pi )}\right\},\;B\left(x\right)=\max\;\left\{\frac{\mathrm{FIR}(A)}{w(A)},\max_{j\in J(x)}B\left(x,j\right)\right\}.$$
  The achieved conditional FIR of each node is backed up according to Equation (16). For an internal node *v*, let $x_{v}=\left(A_{v},U_{v},b_{v}\right)$ be its state, $j_{v}$ the index of its selected test, and $A_{v}$ its candidate fault set. Each node provides a sample $i=(x_{v},j_{v})$, which is stored in the sample repository D together with $\phi (x_{v},j_{v})$, $B(x_{v},j_{v})$, and the completion indicator $h_{i}$. Only when all feasible completions for the state–first-test pair have been enumerated or the archived value reaches the upper bound $u_{j_{v}}\left(x_{v}\right)$ is $h_{i}=1$ assigned; otherwise, $h_{i}=0$. Thus, one tree provides feedback for multiple nodes. In this method, all feedback is backed up together after tree completion.
**(3)**  **Balanced additional completions.** After initial coverage, let $B_{0}\left(x\right)$ be the best achieved conditional value at the sampled state. The eligible first tests are those whose valid upper bounds exceed $B_{0}\left(x\right)$ by the numerical tolerance. This eligibility set and $B_{0}\left(x\right)$ remain fixed throughout the additional-completion stage. The eligible tests are visited cyclically in increasing test-index order, up to a total quota β|J(x)|. This rule balances attempts among the fixed eligible actions. An empty eligible set ends this stage. A feasible root tree attaining the valid root upper bound may end routine completion early. Every achieved subtree is backed up to the archive and label repository.

The network and exploration probability remain fixed within the round. Let *K* be the maximum number of sampled starting states. Exploration decreases linearly with round *k*:

(18) $$\varepsilon_{k}=\varepsilon_{\max}-\left(\varepsilon_{\max}-\varepsilon_{\min}\right)\frac{k-1}{\max\{1,K-1\}}.$$

Maximum completion quotas need not equal realized work because eligibility and valid optimality certificates can stop completion.

### 4.3. Delayed Feedback and Action-Value Network Updates

The action-value network uses a valid return interval and distinguishes achieved feasible lower bounds from certified complete targets. All eligible normalized residuals have equal weight.

**(1)**  **Value interval constraint.** Let the network output be $z_{\theta }\left(\phi \right)\in \left[0,1\right]$, and let the interval width be $r_{j}\left(x\right)=u_{j}\left(x\right)-\ell_{j}\left(x\right)$. The estimated action value of a candidate test is
  (19) $${\hat{Q}}_{\theta }\left(x,j\right)=\ell_{j}\left(x\right)+r_{j}\left(x\right)z_{\theta }\left(\phi \left(x,j\right)\right).$$
**(2)**  **Feedback completion status.** For a state–action sample $i=(x_{v},j_{v})$, write $\phi_{i}=\phi \left(x_{v},j_{v}\right)$, $\ell_{i}=\ell_{j_{v}}\left(x_{v}\right)$, $u_{i}=u_{j_{v}}\left(x_{v}\right)$, and $r_{i}=u_{i}-\ell_{i}=r_{j_{v}}\left(x_{v}\right)$. Construct the error using the latest archived value $B_{i}=B\left(x_{v},j_{v}\right)$. The indicator $h_{i}=1$ means that all feasible completions have been exhaustively searched or the archived value has reached the upper bound; $h_{i}=0$ means that the archive remains a feasible lower bound on the long-term value:
  (20) $$\xi_{i}=\left\{\begin{array}{cc} z_{\theta }\left(\phi_{i}\right)-\left(B_{i}-\ell_{i}\right)/r_{i}, & h_{i}=1(\mathrm{optimum}\;\mathrm{determined}),\\ \min\;\{z_{\theta }\left(\phi_{i}\right)-\left(B_{i}-\ell_{i}\right)/r_{i},\;0\}, & h_{i}=0(\mathrm{optimum}\;\mathrm{not}\;\mathrm{yet}\;\mathrm{determined}). \end{array}\right.$$
  When $r_{i}=0$, the interval value is already determined, and the sample need not be included in network fitting through Equation (20). The achieved archived value, optimal value, and valid bounds satisfy
  $$\max\left\{\ell_{i},B_{i}\right\}\leq Q^{*}\left(x_{v},j_{v}\right)\leq u_{i},\;h_{i}=1⟹B_{i}=Q^{*}\left(x_{v},j_{v}\right).$$
  Complete feedback is used to fit the determined optimal conditional value. Partial feedback is an achieved feasible lower bound, so the one-sided loss corrects predictions below that bound. Certified complete feedback gives a two-sided target. Interval parameterization restricts predictions to valid lower–upper bounds, but a prediction need not be the return of a realizable tree and can exceed the unknown $Q^{*}$. Thus, the current loss does not guarantee elimination of overestimation.
**(3)**  **Unweighted normalized residuals.** Draw a mini-batch from the label repository D, remove duplicate state–action labels from the same original model, and exclude samples with $r_{i}\leq 10^{-12}$ as numerically fixed intervals. Denote the remaining nonempty batch by $D_{\mathrm{batch}}$. The factor $r_{i}$ remains in the target normalization in Equation (20); it is not used as a sample weight. Fault priors still define the terminal MAP objective and the achieved subtree returns. The loss and update are  
  $$\begin{array}{cc} L_{\mathrm{data}}\left(\theta \right) & =\frac{1}{2|D_{\mathrm{batch}}|}\sum_{i\in D_{\mathrm{batch}}}\xi_{i}^{2},\\ L(\theta ) & =L_{\mathrm{data}}\left(\theta \right)+\frac{\kappa }{2}\sum_{l}{∥W_{l}∥}_{F}^{2},\\ g & =\nabla_{\theta }L\left(\theta \right),\;\tilde{g}={g\min\{1,5/\max(∥g∥}_{2},\epsilon_{\mathrm{num}})\},\\ v^{+} & =0.90v+\tilde{g},\;\theta^{+}=\theta -\eta_{\theta }v^{+}. \end{array}$$
  No update is made for an empty eligible batch. The implementation uses $\kappa =10^{-5}$ on weight matrices, momentum 0.90, and global gradient clipping at 5. Biases are not regularized; *v* is the momentum buffer.

### 4.4. Algorithm Workflow and Complexity Analysis

Algorithm 1 summarizes the offline planning procedure for a given MSFG and time budget.

Planning is performed offline for each known MSFG and budget. Two model-derived MAP trees are constructed using immediate and feasible two-step gain per cost. They are retained as candidates for final selection. Only when such a candidate reaches the valid root upper bound is it inserted before routine training, providing a certified shortcut that avoids further completions; eligible candidate feedback can still support at most two network updates. Otherwise, training starts from its own feasible completions. After training, a greedy network tree is added to the archive. Candidates are evaluated using the supplied model, archived subtrees are combined only at matching states (A,U,b), and bounded local search retains improvements. The final DFVL output is the best feasible tree found, rather than necessarily the greedy network tree. Mix1/DIG trees and their returns do not enter this construction.

The final search uses at most two passes, considers three candidate first tests at a visited search state, and allows 100 additional completions. Exact local search is restricted to at most six candidate faults and 3000 expanded nodes overall. Search may stop when a feasible tree attains a valid model-derived upper bound. These are planning limits; no wall-clock cutoff selects the reported trees.

Once a fault occurs, online execution directly uses the tree $\hat{\pi }$ stored offline. Algorithm 2 obtains the response to the test specified at the current node and follows the corresponding branch until it outputs the MAP decision stored at a leaf.

Let $W_{\mathrm{ANN}}$ denote the number of elementary operations in one network forward pass. In a naive implementation, evaluating child-node separability for each candidate test requires scanning the remaining test responses. Sorting the maximum prior probabilities of signature classes introduces an additional logarithmic factor. Including network scoring of candidate actions gives the cost at a single node. Each nonempty binary split adds at least one leaf, so a complete tree contains at most m−1 internal nodes. The time complexities for processing one node and generating the complete tree are therefore

(21) $$\begin{array}{cc} C_{\mathrm{node}} & =O(mn^{2}+nm\log m+nW_{\mathrm{ANN}}),\\ C_{\mathrm{tree}} & =O(m^{2}n^{2}+nm^{2}\log m+mnW_{\mathrm{ANN}}). \end{array}$$

The total offline cost includes model-derived candidate construction, balanced training completions, network updates, and final tree search. If round *k* uses $R_{k}$ completions and $U_{k}$ updates, its cost can be decomposed as

(22) $$C_{\mathrm{off}}=C_{\mathrm{cand}}+\sum_{k=1}^{K_{\mathrm{actual}}}\left(R_{k}C_{\mathrm{tree}}+U_{k}C_{\mathrm{SGD}}\right)+C_{\mathrm{final}}.$$

Here $C_{\mathrm{final}}$ includes network-tree construction, archive combination and bounded local/exact search. Maximum configuration values need not equal realized work. The online representation stores only a test index, two child addresses and a MAP decision per node. Consequently, pure traversal costs O(|ξ|) and compact tree storage costs O(m). The full research archive also contains candidate sets and is distinct from this compact deployment representation.

(23) $$C_{\mathrm{on}}=O(|\xi |),\;|\xi |\leq \min\;\left\{m-1,\left\lfloor \frac{\mathrm{Time}}{\min_{j}c_{j}}\right\rfloor \right\}.$$

**Algorithm 1** DFVL planning for one known MSFG and time budget**Require:** 
Given (D,P,C), budget Time, fixed parameters and seed**Ensure:** 
A stored feasible diagnostic tree $\hat{\pi }$  1:Initialize network θ, archive *B* and label repository D  2:Construct immediate and two-step MAP-gain/cost candidate trees from (D,P,C,Time)  3:If a candidate attains the root upper bound, record it; use eligible certified feedback for at most two network updates and skip routine completions  4:**if** no initial candidate certifies the optimum **then**  5:    **for** k=1,…,K **do**  6:        Sample a root or reachable intermediate state x=(A,U,b)  7:        Compute feasible first tests and valid value bounds; freeze $\theta ,\varepsilon_{k}$  8:        Collect up to $N_{\mathrm{init}}$ completions per feasible first test, stopping if the root optimum is certified  9:        Back up each feasible tree to update *B* and state–action labels in D10:        Freeze $B_{0}\left(x\right)$ and eligible first tests; set quota β|J(x)|11:        **for** each permitted balanced completion **do**12:           Visit the eligible tests cyclically in increasing index order; stop if none remain13:           Complete the selected first test; back up feasible feedback and labels14:           **if** a root tree attains the valid upper bound **then**15:               Stop routine completion16:           **end if**17:        **end for**18:        Mark feedback complete only when proved by exhaustive search or a valid upper bound19:        Update the network from eligible archived labels after all current-round feedback20:        **if** the root optimum has been certified **then**21:           **break**22:        **end if**23:    **end for**24:**end if**25:Build a greedy network tree; add it and the model-derived candidates to the archive26:Combine archived subtrees only at matching (A,U,b) states27:Perform bounded local search and small-substate exact search, retaining feasible improvements28:Select the largest-FIR feasible tree found; ties prefer smaller maximum path duration29:Store test indices, child addresses and terminal MAP decisions

**Algorithm 2** Online fault diagnosis along a pregenerated diagnostic tree
**Require:** 
Stored diagnostic tree $\hat{\pi }$**Ensure:** 
Diagnosis $\hat{s}$, executed test sequence E, and cumulative duration *t*1:$v\leftarrow \mathrm{root}(\hat{\pi })$; t←0; E←()2:**while***v* is an internal node **do**3:    Read from *v* the stored test index *j*; execute $t_{j}$ and wait for completion4:    Obtain the test response y∈{0,1}; $t\leftarrow t+c_{j}$5:    Append $t_{j}$ to E; v←child(v,y)6:
**end while**
7:$\hat{s}\leftarrow v$’s stored MAP fault index8:
**return** $\left(\hat{s},E,t\right)$




## 5. Experimental Validation

### 5.1. Experimental Setup

The evaluations comprise simulations and an engineering-model calculation for a power-grid measurement device. Simulations compare FIR across problem sizes and time budgets. The engineering case considers a single acquisition resource and a limited diagnostic window. FIR is the sole performance measure; timings and storage describe implementation cost. Calculations used MATLAB R2018b on 64-bit Windows 11 Pro, with an Intel Core i5-12400 processor and approximately 16 GB RAM.

DFVL is trained separately for each supplied D/P/C model and time budget, including the device model. Two training settings were compared: K=12 with at most two updates per eligible round, and K=32 with at most eight updates per eligible round. The 18 model–budget conditions comprised three sizes (m=n=30,60,100), two models per size, and three relative budgets (α=0.20,0.26,0.40). These conditions form a subset of the formal evaluation. Both settings achieved a mean full-pipeline FIR of 77.09%; the $10^{-10}$ tie rule selected the smaller setting, which was then fixed for the formal evaluations. The final parameters are listed in Table 1, and state sampling and stopping rules are specified in Section 4.2.

Mix1 and DIG receive the same D,P,C and use the same terminal MAP decisions as DFVL. DIG uses node-conditional probabilities $q_{i}=p_{i}/w\left(A\right)$ and $q_{0}=w\left(A_{j}^{0}\right)/w\left(A\right)$, with score $\left[-q_{0}\log_{2}q_{0}-\sum_{i\in A_{j}^{1}}q_{i}\log_{2}q_{i}\right]/c_{j}$ and $0\log_{2}0=0$; tied scores select the smallest test index. Budget-aware variants first exclude tests that cannot finish. Two further references maximize immediate MAP gain per cost and feasible two-step MAP gain per first-test cost; they also supply DFVL’s model-derived candidate trees.

The independent sampling unit is the D/P/C model. Overall comparisons average the 12 paired budget differences within each of the 90 models before constructing paired *t* intervals (89 degrees of freedom) and two-sided exact sign tests, with Holm adjustment for the two DFVL–baseline comparisons. Pointwise intervals use ten models (9 degrees of freedom), conditional on recorded algorithm seeds. Repeated budgets or seeds are not independent model replicates.

### 5.2. Simulation Experiments

This subsection examines the effects of problem size and time budget on algorithm performance. Nine sizes, m=n=20,30,…,100, were considered, with 10 independently generated MSFG instances for each size. For each instance, all algorithms use the same *D*, *P*, *C*, and actual time budget, and their mean FIR is compared at different budget levels.

The simulation data were generated as follows. The entries of *D* are independently Bernoulli distributed. Faults are assigned to grades B, C, and D in specified count ratios, and the grade weights in Section 2.3 are normalized to obtain *P*. Tests are assigned to three duration levels in specified count ratios to obtain *C*. The same distribution parameters and grade ratios are used for all sizes. When the specified ratios do not yield integer counts, the largest remainder method is used. The settings are listed in Table 2.

For comparison baseline *a*, let $\pi_{a}^{\mathrm{ref}}$ denote its complete diagnostic tree, and let $P(\pi_{a}^{\mathrm{ref}})$ denote its set of root-to-leaf paths. For each instance, the common reference time is the maximum path duration among the Mix1 and DIG trees. The budget at each level is defined as

(24) $$\begin{array}{cc} T_{\max} & =\max_{a\in \{\mathrm{Mix}1,\mathrm{DIG}\}}\left\{\max_{\xi \in P(\pi_{a}^{\mathrm{ref}})}\sum_{t_{j}\in \xi }c_{j}\right\},\\ \mathrm{Time} & =\alpha T_{\max}. \end{array}$$

For each model, Mix1 and DIG retain their complete-tree test order. The next response is included only when its test finishes by the deadline; equality is included within the numerical tolerance $10^{-10}\max\left\{1,\sum_{j}c_{j}\right\}$. If the next test cannot finish, the strategy stops at the current node and makes its stored MAP decision; it does not skip to another test. DFVL instead plans a feasible tree directly under the same budget. Zero and repeated response rows are retained for every method.

**Figure 3:**
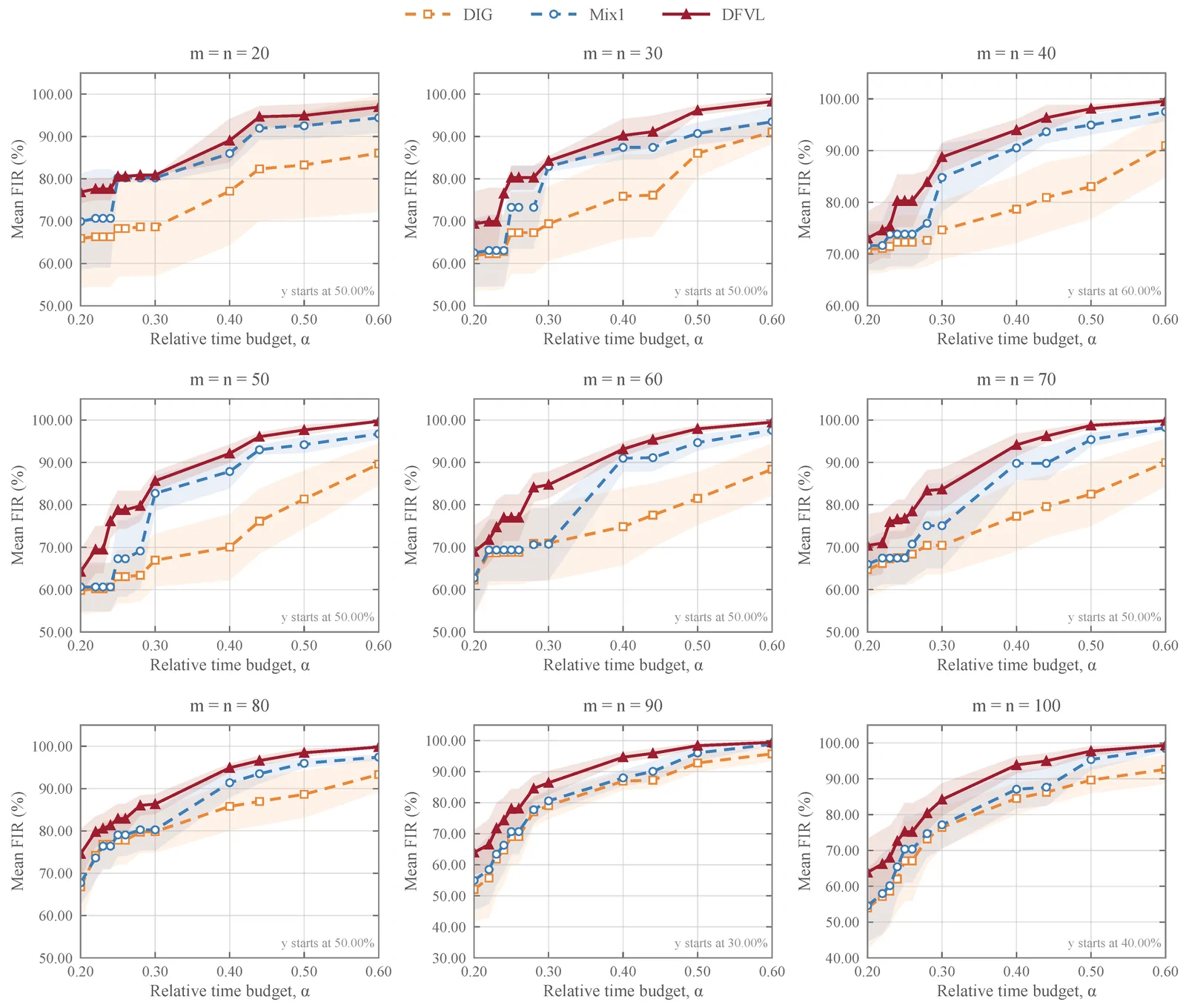
Mean terminal MAP FIR at nine problem sizes and twelve relative budgets. Lines connect the observed means across ten independent D/P/C models per size. Shaded bands are pointwise 95% *t* intervals (n=10, 9 degrees of freedom), conditional on the recorded algorithm seeds. Methods share the model inputs and deadline $\alpha T_{\max}$.

For each problem size, the result at each budget level is the mean over the 10 instances:

(25) $${\bar{\mathrm{FIR}}}_{a}\left(\alpha \right)=\frac{1}{10}\sum_{s=1}^{10}{\mathrm{FIR}}_{a,s}\;\left(\alpha T_{\max}^{\left(s\right)}\right),$$

where *a* and *s* index the algorithm and instance, respectively. At zero budget, the initial MAP decision is retained. If *D* contains identical fault-response rows, the corresponding indistinguishable ambiguity sets are retained. Figure 3 presents the results for the nine sizes; the horizontal and vertical axes in each panel show α and mean FIR, respectively.

**(1)**  **Effect of the time budget.** As shown in Figure 3, the mean FIR of each algorithm generally increases in steps with α at all nine sizes. At small budgets, whether a test finishes before the deadline directly changes the terminal ambiguity sets, producing plateaus and increases in the curves. As the budget increases, more tests can be completed, and the isolation rates of the three methods gradually increase. Separate finite planning runs at different budgets need not return nested DFVL trees, so its observed curve need not be monotone.
**(2)**  **Effect of problem size.** From 20×20 to 100×100, the differences between algorithms vary with both problem size and budget. DFVL and Mix1 differ more clearly under some tight-budget conditions, while Mix1 approaches DFVL over some intervals at the larger sizes. Changing the problem size alters the possible fault partitions and time allocations; consequently, the differences in isolation gain do not simply increase monotonically with the number of faults or tests.
**(3)**  **Performance differences between algorithms.** Across the 1080 model–budget conditions, mean FIR was 83.53% for DFVL, 77.96% for Mix1 and 73.24% for DIG. The model-level mean paired gains were 5.58 percentage points over Mix1 (95% interval 4.88–6.27) and 10.29 over DIG (8.79–11.80), with Holm-adjusted $p=3.23\times 10^{-27}$ for both comparisons. The budget-aware Mix1 and DIG means were 77.96% and 73.58%; the two-step and immediate MAP references gave 82.47% and 81.58%. These comparisons evaluate the complete hybrid DFVL procedure, which combines value learning, historical-tree retention, model-derived MAP candidates, and bounded search.

The Bernoulli (0.50) simulations are controlled randomized comparisons; they do not reproduce the sparse response structure or fault-grade composition of the device model.

**Retained-history ablation.** The control starts from the same learned prefix, network, feedback labels, and random-generator state as DFVL. Before final search, only the control clears inherited feasible-tree entries and removes the root archived candidate. The two MAP candidates, current network tree, subsequent new archiving and final-search limits are retained. This comparison evaluates the joint effect of retaining historical feasible trees and their archived root candidate.

**Figure 4:**
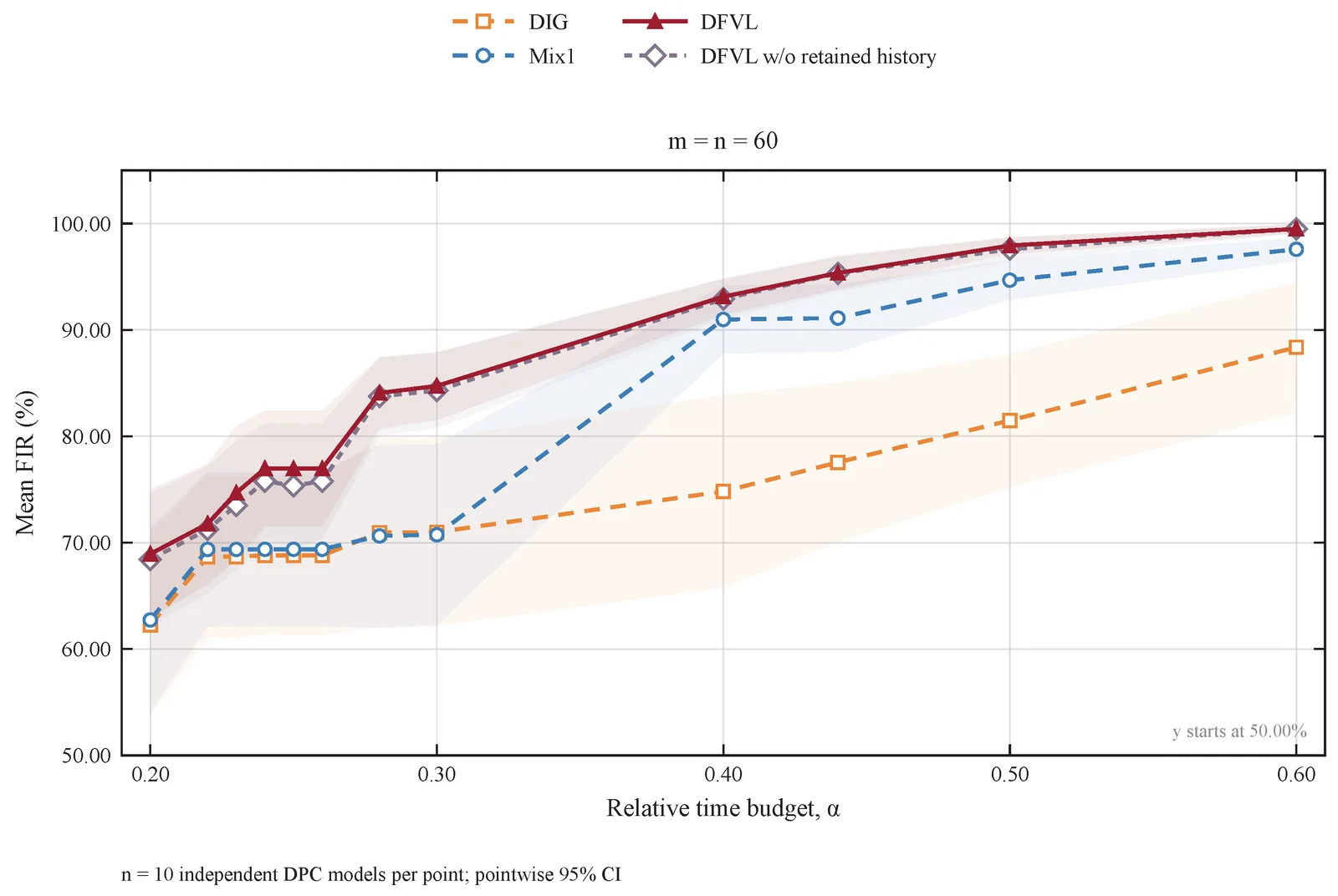
Four-strategy retained-history ablation on ten 60×60 models at twelve relative budgets. Lines connect the observed means; shading gives pointwise 95% *t* intervals across the ten models (9 degrees of freedom), conditional on recorded seeds. The four strategies use the same D,P,C and deadline $\alpha T_{\max}$. The shading colors match the curves: orange for DIG, blue for Mix1, red for DFVL, and purple for DFVL without retained history.

Figure 4 compares Mix1, DIG, DFVL and this control on all ten 60×60 models at all twelve budgets, giving 120 paired conditions. After averaging the twelve paired differences within each model, the mean DFVL-minus-control difference was 0.63 percentage points (95% *t* interval [−0.01,1.27], ten models, 9 degrees of freedom). The condition-level win/tie/loss count was 23/95/2. The interval includes zero, and the observed effects vary across models and budgets.

On 30 independent 8×8 models at an absolute budget of 6, DFVL attained the independent Bellman optimum in all cases: mean FIR 93.41%, versus 93.07% for Mix1 and 92.43% for DIG. The exact solutions were not supplied to DFVL.

Three-seed repeats on twenty additional 60×60 models at α∈{0.20,0.26,0.40} gave a mean within-model/budget FIR sample SD of 0.13 percentage points (maximum 1.96); 45 of 60 groups tied across seeds.

At m=n=60 and α=0.20, changing $\varepsilon_{\max}$ from 0.30 to 0.15 or β from 2 to 1 left all ten matched FIR values unchanged (mean 68.94%). This local check describes the stated setting.

### 5.3. Engineering-Model Evaluation of a Power-Grid Measurement Device

**Figure 5:**
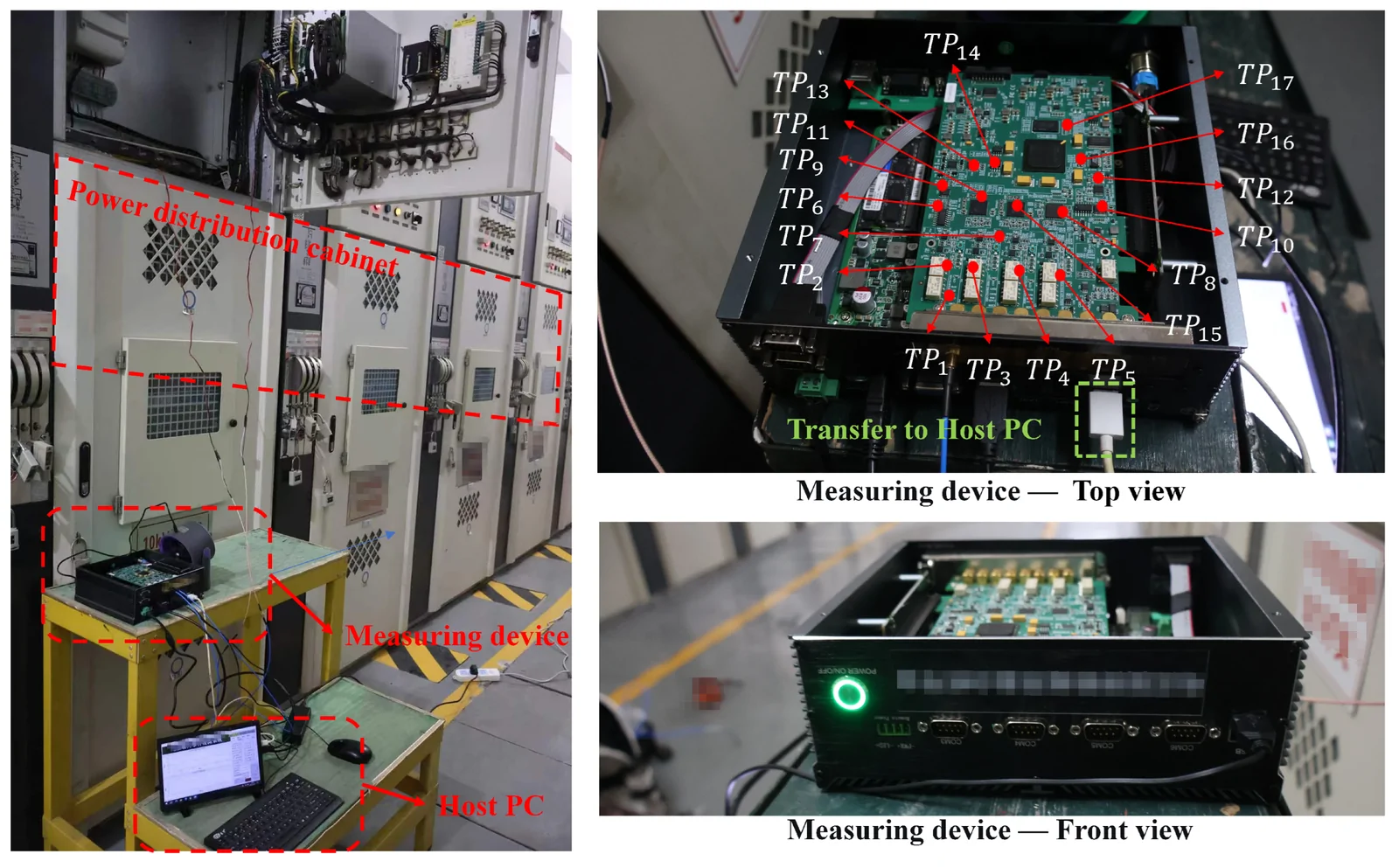
Measurement device and existing test access used to define the engineering model.

An engineering-model calculation was performed for a μPMU measurement device installed in a distribution cabinet; the platform is shown in Figure 5. The reported FIR values are calculated from supplied D/P/C inputs, not hardware fault-injection outcomes. This device is susceptible to faults during continuous operation, so BIT must be incorporated at the design stage to support fault detection and diagnosis during service. The device continuously acquires and analyzes current, voltage, and phasor data. Fault diagnosis uses measurements obtained under load and the relationships among them. Once the device is taken offline or unloaded, the response data for the corresponding load conditions are unavailable, and the criteria for the relevant in-service tests lose their required data basis. Therefore, faults within the device must be diagnosed during normal load operation.

Because of space and resource constraints, the device uses a single-channel acquisition card connected to different test points through multiplexed switching. Switching during acquisition affects the waveform being recorded. Each test must therefore complete the prescribed acquisition interval and decision process before the system switches to the next test point. Tests require different numbers of waveform cycles; together with switching, settling, and analysis times, these requirements produce different execution costs. BIT therefore uses sequential tests that share a single acquisition resource.

The internal diagnostic window is Time=36 in normalized units; its physical conversion is not disclosed. Costs 1,3,9 use the same normalization and include switching, settling, acquisition, analysis, and diagnostic decision time.

The fixed model contains 57 faults, 43 tests and 18 test points. The supplied fault grades contain 19, 23 and 15 faults, with weights 100,10,1 and a total weight of 2145. The cost levels contain 8, 30 and 5 tests. Input elicitation and inter-expert agreement are outside this strategy-generation study. Appendix A provides every model entry and the fault/test definitions.

There are 176 nonzero entries in *D*, a density of 7.18%. The existing test access produces nine zero-response rows ($s_{8},s_{9},s_{10},s_{14},s_{15},s_{22},s_{43},s_{52},s_{53}$), with a total weight of 441, and five additional repeated-signature pairs. All faults are retained. The 44 response-equivalence classes give an unlimited-time MAP ceiling of 1673/2145 (78.00%). These are coverage and discrimination limits of the fixed test-point layout.

**Figure 6:**
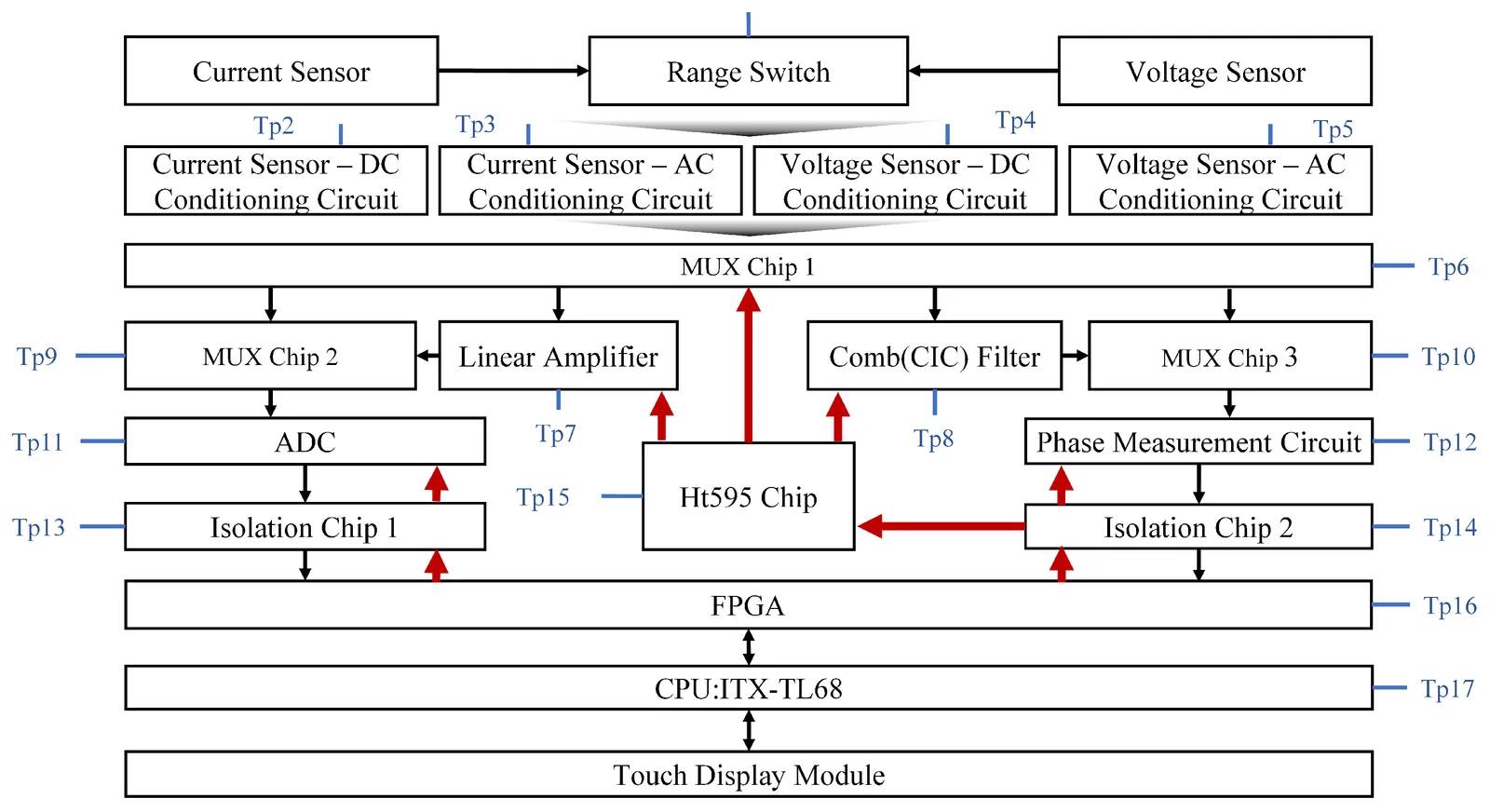
Multi-signal flow graph and available test points of the measurement device. The red arrows indicate the data bus.

Figure 6 shows the multi-signal flow graph and available test points of the measurement device.

Table 3 compares the four strategies at normalized budgets 12, 24, and 36. The retained-history control uses the matched-prefix definition in Section 5.2.

At Time 36, DFVL gave FIR 75.90%, versus 53.15% for Mix1 and 61.86% for DIG. Their correct-decision weights were 1628, 1140, and 1327 out of 2145, respectively. DFVL remained 2.10 percentage points below the unlimited-time ceiling. Historical-tree retention increased FIR by 3.17, 6.48 and 9.18 percentage points at budgets 12, 24 and 36, respectively. Appendix B shows the three nominal-Time36 trees.

**Prior sensitivity.** With *D*, *C*, and Time = 36 unchanged, all four strategies were regenerated using uniform priors $p_{i}=1/57$, and DFVL was retrained for this model and budget. FIR was 59.65% for Mix1 and DIG, 66.67% for DFVL and 59.65% for the control. The corresponding unlimited-time ceiling was 44/57 (77.19%).

**Implementation resources.** Nominal-Time36 offline planning took 180.46 s: candidate construction 0.11 s, training 178.45 s, network-tree construction $5.80\times 10^{-3}$ s and final search 1.89 s. Training included 170.19 s of completion and 0.27 s of batch updates. The 69-node deployment table used 1104 B (494 B serialized); the network object used 26,840 B, or 53,656 B with optimizer buffers. After one untimed traversal of all 57 fault cases, software traversal was measured using MATLAB’s tic/toc functions over five batches, each repeating the 57 cases 1000 times. Traversal used the preconstructed deployment table and deterministic responses stored in *D*. The mean batch duration divided by 57,000 was $2.14\times 10^{-7}$ s/path. These are software timing and object-size measurements, not peak process RAM or a conversion of normalized Time to physical seconds. Decision time is already included in *C*.

## 6. Conclusions

Traditional offline methods generally optimize diagnostic cost while satisfying isolation requirements, rather than directly optimizing the time-constrained FIR defined in this study. To address this problem, an online TSG optimization formulation was established using an MSFG. DFVL combines value recursion and delayed feasible-subtree feedback with historical-tree retention, model-derived MAP candidates, and bounded search. Balanced completions and unweighted normalized residuals define its sampling and learning rules.

Across nine problem sizes and twelve budgets, mean FIR was 83.53% for DFVL, 77.96% for Mix1 and 73.24% for DIG. At a normalized device budget of 36, the respective FIR values were 75.90%, 53.15% and 61.86%. Historical-tree retention improved device FIR by 3.17–9.18 percentage points; its model-averaged simulation effect was 0.63 points, with a 95% interval spanning zero.

The study assumes one listed fault at a time, given D/P/C, deterministic responses, and fixed durations. Each model and budget requires its own offline planning. The device results are model calculations, not field diagnostic accuracy. The effects of test errors and duration fluctuations on strategy performance remain to be investigated. Future work will incorporate unreliable tests and random durations and evaluate the method in more complex engineering systems.

## Figures and Tables

**Table 1:** Experimental settings for the algorithms.

Algorithm	Main Parameters and Training Settings
DFVL	Network 16→64→32→1, ReLU hidden and Sigmoid output; K=12, $N_{\mathrm{init}}=2$, β=2, at most two updates per eligible round; $\varepsilon_{\max}=0.30$, $\varepsilon_{\min}=0.05$; balanced additional completions and unit-weight normalized residuals; learning rate 0.01, batch 256, momentum 0.90, $\kappa =10^{-5}$, gradient clip 5; final limits: two passes, width 3, 100 completions, local exact states with at most six faults and 3000 node expansions overall
Mix1	Mixing weight $\alpha_{\mathrm{Mix}1}\left(\rho_{D}\right)=0.01+0.67/\left[1+{\left(\rho_{D}/0.40\right)}^{10}\right]$, where $\rho_{D}=\sum_{i}\sum_{j}d_{ij}/\left(mn\right)$ is the empirical proportion of entries in the initial *D* matrix that equal 1
DIG	Node-conditional information score per cost, defined in the text; deterministic lowest-index tie breaking

**Table 2:** Simulation data and time budget settings.

Setting	Value
Numbers of faults and tests	m=n=20,30,…,100
Instances per problem size	10
Bernoulli parameter $\mathrm{Pr}(d_{ij}=1)$	0.50
Prior weights of individual faults in the three grades	100:10:1
Fault count ratio across the three grades	2:5:10
Test durations at the three levels	1, 3, and 10
Test count ratio across the three duration levels	1:5:10
Common reference time $T_{\max}$	Maximum path duration among the trees of the two comparison baselines, Mix1 and DIG
Relative time budget levels α	0.20,0.22,0.23,0.24,0.25,0.26, 0.28,0.30,0.40,0.44,0.50,0.60

**Table 3:** Model-based FIR under nominal priors at three normalized device budgets.

Time	Mix1 (%)	DIG (%)	DFVL (%)	DFVL w/o Retained History (%)
12	15.43	19.63	34.08	30.91
24	38.51	42.66	57.11	50.63
36	53.15	61.86	75.90	66.71

**Figure A1:**
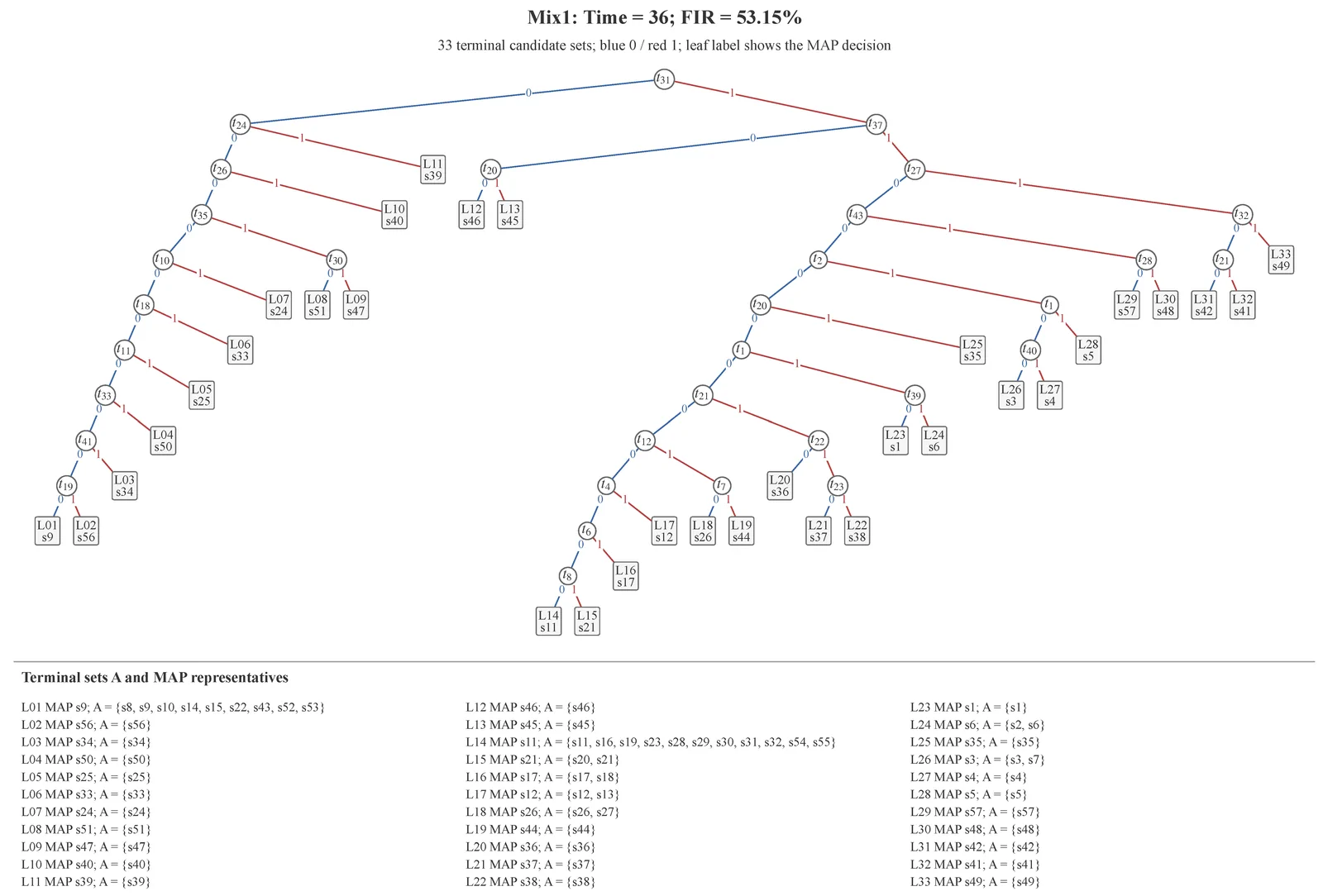
Mix1 diagnostic tree and node candidate sets at normalized Time=36.

**Figure A2:**
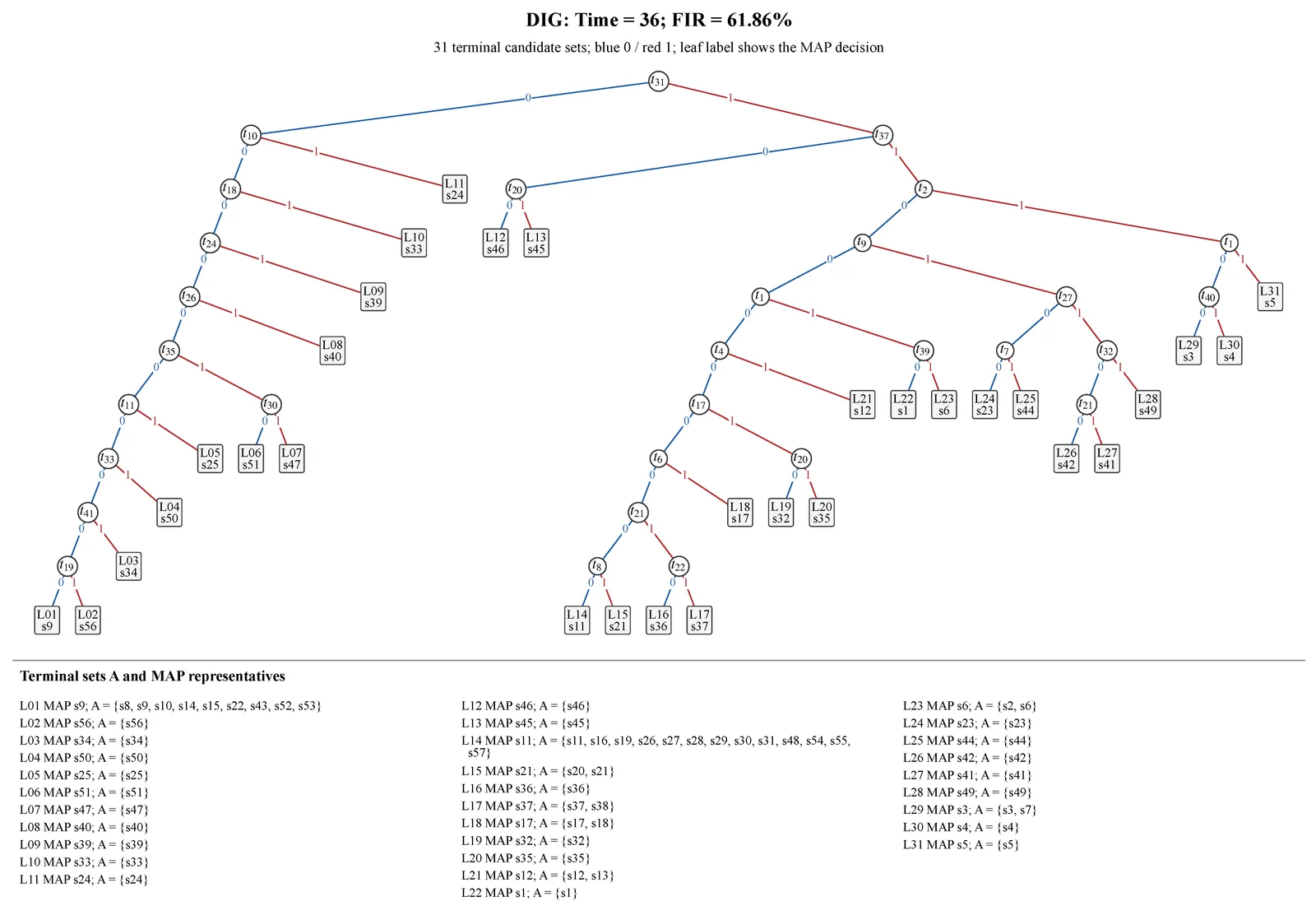
DIG diagnostic tree and node candidate sets at normalized Time=36.

**Figure A3:**
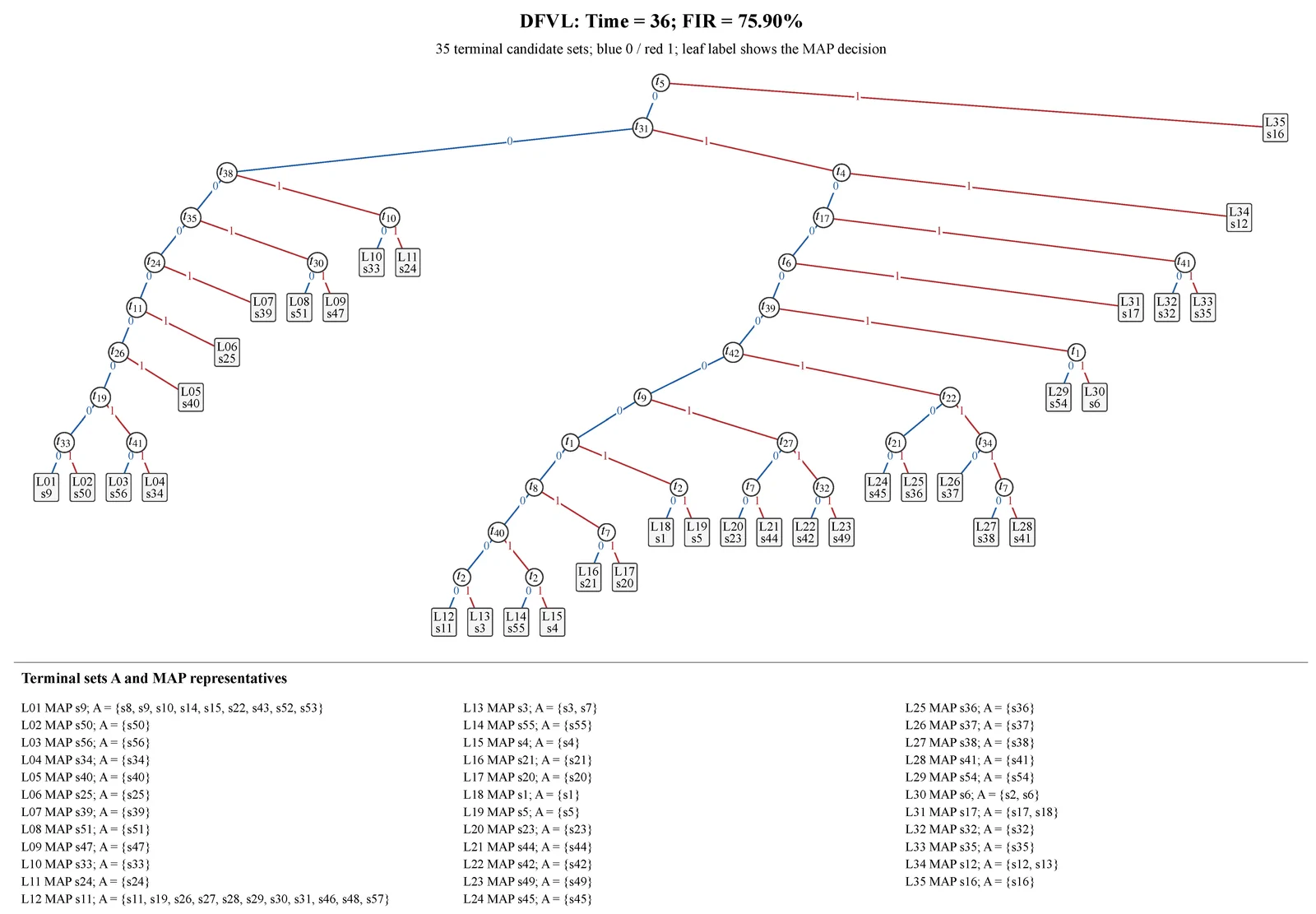
DFVL diagnostic tree and node candidate sets at normalized Time=36.

## Data Availability

The measurement-device model parameters and diagnostic trees are provided in Appendix A and Appendix B.

## Appendix A. Complete MSFG Parameters of the Measurement Device

The fault list and the online test list are given in Table A1 and Table A2, respectively. Table A3 provides the complete response matrix, prior weights, and test costs, where $w_{i}$ is the unnormalized prior weight of fault $s_{i}$ and the prior probability is calculated as $p_{i}=w_{i}/2145$.

**Table A1:** Complete fault list of the measurement device.

ID	Component or Function	Fault Mode	Grade
$s_{1}$	M1 Current sensor	Drift in current sensor sensitivity gain	B
$s_{2}$	M1 Current sensor	Open-circuit current sensor output or no valid signal	D
$s_{3}$	M2 Voltage sensor	Drift in voltage sensor transformation-ratio gain	B
$s_{4}$	M2 Voltage sensor	Open-circuit voltage sensor output or no valid signal	D
$s_{5}$	M3 Range switching module	Selected range differs from the target range	C
$s_{6}$	M3 Range switching module	Failure of conduction through the current range channel	C
$s_{7}$	M3 Range switching module	Abnormal contact impedance in the voltage range channel	B
$s_{8}$	M3 Range switching module	Excessive range switching settling time	C
$s_{9}$	M4 Current DC conditioning circuit	DC conditioning gain out of calibration	B
$s_{10}$	M4 Current DC conditioning circuit	DC conditioning zero-point offset	B
$s_{11}$	M5 Current AC conditioning circuit	AC conditioning passband gain out of calibration	B
$s_{12}$	M5 Current AC conditioning circuit	AC conditioning phase shift	B
$s_{13}$	M5 Current AC conditioning circuit	Reduced AC conditioning bandwidth	C
$s_{14}$	M6 Voltage DC conditioning circuit	DC conditioning gain out of calibration	B
$s_{15}$	M6 Voltage DC conditioning circuit	DC conditioning zero-point offset	B
$s_{16}$	M7 Voltage AC conditioning circuit	AC conditioning passband gain out of calibration	B
$s_{17}$	M7 Voltage AC conditioning circuit	AC conditioning phase shift	B
$s_{18}$	M7 Voltage AC conditioning circuit	Reduced AC conditioning bandwidth	C
$s_{19}$	M8 MUX 1	Incorrect correspondence between the selection address and the intended channel	C
$s_{20}$	M8 MUX 1	Open circuit or stuck-off condition in the selected channel	D
$s_{21}$	M8 MUX 1	Amplitude error caused by on-resistance	B
$s_{22}$	M8 MUX 1	Crosstalk from unselected channels exceeds the limit	C
$s_{23}$	M9 Linear amplifier	Closed-loop gain coefficient out of calibration	B
$s_{24}$	M9 Linear amplifier	DC offset at the output	B
$s_{25}$	M9 Linear amplifier	Limited output swing causing clipping of the operating waveform	C
$s_{26}$	M10 Comb (CIC) filter module	Filter magnitude response differs from the specified response	C
$s_{27}$	M10 Comb (CIC) filter module	Passband group delay differs from the specified value	C
$s_{28}$	M11 MUX 2	Incorrect selection between the pass-through and amplified branches	C
$s_{29}$	M11 MUX 2	Open circuit in the selected output channel	D
$s_{30}$	M12 MUX 3	Incorrect selection between the pass-through and filtered branches	C
$s_{31}$	M12 MUX 3	Open circuit in the selected output channel	D
$s_{32}$	M13 ADC	Abnormal ADC reference scale or conversion gain	B
$s_{33}$	M13 ADC	ADC zero-code offset	B
$s_{34}$	M13 ADC	Stuck data bit within the current operating ADC code range	D
$s_{35}$	M13 ADC	Missing samples caused by loss of conversion triggers	C
$s_{36}$	M14 Phase measurement circuit	Fixed phase measurement bias	B
$s_{37}$	M14 Phase measurement circuit	Incorrect edge polarity selection	D
$s_{38}$	M14 Phase measurement circuit	Edge counting or result updates stalled	D
$s_{39}$	M15 Isolation chip 1	Bit errors caused by timing distortion in the isolated data channel	C
$s_{40}$	M16 Isolation chip 2	Bit errors in the isolated transmission of phase results	C
$s_{41}$	M16 Isolation chip 2	Loss of valid pulses in reverse control transmission	C
$s_{42}$	M17 Ht595 control chip	Control output state not updated in response to issued commands	C
$s_{43}$	M17 Ht595 control chip	Control output update delay exceeds the limit	C
$s_{44}$	M17 Ht595 control chip	Control output enable failure resulting in a high-impedance state	D
$s_{45}$	M18 FPGA	Sampling clock division ratio differs from the configured value	D
$s_{46}$	M18 FPGA	Misaligned output-frame channel identifiers or sequence numbers	D
$s_{47}$	M18 FPGA	Frame loss in the continuous acquisition buffer	C
$s_{48}$	M18 FPGA	Acquisition data output channel stalled	D
$s_{49}$	M19 CPU (ITX-TL68)	Acquisition routing configuration not updated	C
$s_{50}$	M19 CPU (ITX-TL68)	Incorrect scaling conversion of CPU phasor amplitudes	C
$s_{51}$	M19 CPU (ITX-TL68)	Result record updates stalled or old frames repeated	C
$s_{52}$	M20 Touch display module	No data returned by the touch interface	D
$s_{53}$	M20 Touch display module	Touch coordinate calibration offset	C
$s_{54}$	M1 Current sensor	Fundamental phase shift of the current sensor	B
$s_{55}$	M2 Voltage sensor	Fundamental phase shift of the voltage sensor	B
$s_{56}$	M13 ADC	Local ADC nonlinearity exceeds the limit within the operating code range	D
$s_{57}$	M18 FPGA	Incorrect FPGA sampling word length or sign-extension configuration	D

**Table A2:** Complete online test list of the measurement device.

ID	In-Service Comparison Reference	Measurement Point	Online Test
$t_{1}$	Synchronized clamp-on current reference	$Tp_{1}$	Comparison of current amplitude in the range in use with an independent reference
$t_{2}$	Synchronized high-impedance voltage reference	$Tp_{1}$	Comparison of voltage amplitude in the range in use with an independent reference
$t_{3}$	$Tp_{1}$	$Tp_{3}$	Comparison of current AC conditioning gain at the fundamental frequency
$t_{4}$	$Tp_{1}$	$Tp_{3}$	Comparison of current AC conditioning phase at the fundamental frequency
$t_{5}$	$Tp_{1}$	$Tp_{5}$	Comparison of voltage AC conditioning gain at the fundamental frequency
$t_{6}$	$Tp_{1}$	$Tp_{5}$	Comparison of voltage AC conditioning phase at the fundamental frequency
$t_{7}$	$Tp_{2}$,$Tp_{3}$,$Tp_{4}$,$Tp_{5}$; Current configuration record	$Tp_{6}$	Correspondence between the MUX1 address in use and the selected waveform
$t_{8}$	$Tp_{2}$,$Tp_{3}$,$Tp_{4}$,$Tp_{5}$; Current configuration record	$Tp_{6}$	Amplitude transfer residual of the selected MUX1 channel
$t_{9}$	$Tp_{6}$	$Tp_{7}$	Comparison of the linear amplifier closed-loop gain during operation
$t_{10}$	$Tp_{6}$	$Tp_{7}$	Linear amplifier offset residual during operation
$t_{11}$	$Tp_{6}$	$Tp_{7}$	Linear amplifier waveform clipping residual during operation
$t_{12}$	$Tp_{6}$	$Tp_{8}$	Filter module fundamental phase residual during operation
$t_{13}$	$Tp_{6}$,$Tp_{7}$; Current configuration record	$Tp_{9}$	Correspondence of the MUX2 pass-through or amplified path in use
$t_{14}$	$Tp_{6}$,$Tp_{7}$; Current configuration record	$Tp_{9}$	Transfer continuity of the MUX2 channel in use
$t_{15}$	$Tp_{6}$,$Tp_{8}$; Current configuration record	$Tp_{10}$	Correspondence of the MUX3 pass-through or filtered path in use
$t_{16}$	$Tp_{6}$,$Tp_{8}$; Current configuration record	$Tp_{10}$	Transfer continuity of the MUX3 channel in use
$t_{17}$	$Tp_{9}$	$Tp_{11}$	Gain regression between the ADC input and code values during operation
$t_{18}$	$Tp_{9}$	$Tp_{11}$	Intercept residual between the ADC input and code values during operation
$t_{19}$	$Tp_{9}$	$Tp_{11}$	Per-sample residual within the current operating ADC code range
$t_{20}$	$Tp_{9}$	$Tp_{11}$	ADC conversion timing and sample counts during operation
$t_{21}$	$Tp_{10}$	$Tp_{12}$	Comparison of phase measurement edges and results during operation
$t_{22}$	$Tp_{10}$	$Tp_{12}$	Correspondence of phase measurement edge polarity during operation
$t_{23}$	$Tp_{10}$	$Tp_{12}$	Phase measurement frequency and result updates during operation
$t_{24}$	$Tp_{11}$	$Tp_{13}$	Bit-by-bit comparison of actual data across isolation chip 1
$t_{25}$	$Tp_{11}$	$Tp_{13}$	Comparison of actual transmission delay across isolation chip 1
$t_{26}$	$Tp_{12}$	$Tp_{14}$	Bit-by-bit comparison of actual phase data across isolation chip 2
$t_{27}$	$Tp_{14}$; Current valid configuration record	$Tp_{15}$	Consistency of the Ht595 control state in use
$t_{28}$	$Tp_{13}$,$Tp_{14}$	$Tp_{16}$	Correspondence of FPGA channel identifiers and frame numbers during operation
$t_{29}$	$Tp_{13}$,$Tp_{14}$	$Tp_{16}$	FPGA sampling timestamp intervals during operation
$t_{30}$	$Tp_{13}$,$Tp_{14}$	$Tp_{16}$	Completeness of consecutive FPGA frames during operation
$t_{31}$	Synchronized current/voltage bypass reference	$Tp_{16}$	Comparison of FPGA amplitude and phase during operation with the bypass reference
$t_{32}$	Current valid configuration record	$Tp_{17}$	Consistency of CPU configuration messages in use
$t_{33}$	$Tp_{16}$	$Tp_{17}$	Independent recalculation of phasors from CPU operating data
$t_{34}$	$Tp_{16}$	$Tp_{17}$	CPU operating result update times and old-frame detection
$t_{35}$	$Tp_{16}$	$Tp_{17}$	Consistency check of CPU and FPGA frames during operation
$t_{36}$	$Tp_{16}$	$Tp_{17}$	Independent recalculation of CPU active power results during operation
$t_{37}$	Synchronized current/voltage bypass reference	$Tp_{17}$	Comparison of the complex power vector during operation with the bypass reference
$t_{38}$	Synchronized current/voltage bypass reference	$Tp_{16}$	Residual between the FPGA waveform mean during operation and the reference
$t_{39}$	Synchronized clamp-on current phase reference	$Tp_{1}$	Comparison of the fundamental phase of the current sensor chain in use
$t_{40}$	Synchronized high-impedance voltage phase reference	$Tp_{1}$	Comparison of the fundamental phase of the voltage sensor chain in use
$t_{41}$	$Tp_{9}$	$Tp_{11}$	Verification of ADC data-bit activity during operation
$t_{42}$	$Tp_{10}$; Normal phase reference/synchronized time base	$Tp_{14}$	Comparison of phase results after isolation with edges during operation
$t_{43}$	$Tp_{13}$	$Tp_{16}$	Verification of FPGA sampling payload encoding during operation

**Table A3:** Complete response matrix *D*, prior weights $w_{i}$, and test costs *C* of the measurement device.

Fault	$w_{i}$	$t_{1}$	$t_{2}$	$t_{3}$	$t_{4}$	$t_{5}$	$t_{6}$	$t_{7}$	$t_{8}$	$t_{9}$	$t_{10}$	$t_{11}$	$t_{12}$	$t_{13}$	$t_{14}$	$t_{15}$	$t_{16}$	$t_{17}$	$t_{18}$	$t_{19}$	$t_{20}$	$t_{21}$	$t_{22}$	$t_{23}$	$t_{24}$	$t_{25}$	$t_{26}$	$t_{27}$	$t_{28}$	$t_{29}$	$t_{30}$	$t_{31}$	$t_{32}$	$t_{33}$	$t_{34}$	$t_{35}$	$t_{36}$	$t_{37}$	$t_{38}$	$t_{39}$	$t_{40}$	$t_{41}$	$t_{42}$	$t_{43}$
Cost	—	3	3	3	3	3	3	3	3	3	3	3	3	3	3	3	3	3	3	9	1	3	3	3	1	3	1	1	1	1	9	3	1	3	9	3	3	9	9	3	3	3	3	1
$s_{1}$	100	1	0	0	0	0	0	0	0	0	0	0	0	0	0	0	0	0	0	0	0	0	0	0	0	0	0	0	0	0	0	1	0	0	0	0	0	1	0	0	0	0	0	0
$s_{2}$	1	1	0	0	0	0	0	0	0	0	0	0	0	0	0	0	0	0	0	0	0	0	0	0	0	0	0	0	0	0	0	1	0	0	0	0	0	1	0	1	0	0	0	0
$s_{3}$	100	0	1	0	0	0	0	0	0	0	0	0	0	0	0	0	0	0	0	0	0	0	0	0	0	0	0	0	0	0	0	1	0	0	0	0	0	1	0	0	0	0	0	0
$s_{4}$	1	0	1	0	0	0	0	0	0	0	0	0	0	0	0	0	0	0	0	0	0	0	0	0	0	0	0	0	0	0	0	1	0	0	0	0	0	1	0	0	1	0	0	0
$s_{5}$	10	1	1	0	0	0	0	0	0	0	0	0	0	0	0	0	0	0	0	0	0	0	0	0	0	0	0	0	0	0	0	1	0	0	0	0	0	1	0	0	0	0	0	0
$s_{6}$	10	1	0	0	0	0	0	0	0	0	0	0	0	0	0	0	0	0	0	0	0	0	0	0	0	0	0	0	0	0	0	1	0	0	0	0	0	1	0	1	0	0	0	0
$s_{7}$	100	0	1	0	0	0	0	0	0	0	0	0	0	0	0	0	0	0	0	0	0	0	0	0	0	0	0	0	0	0	0	1	0	0	0	0	0	1	0	0	0	0	0	0
$s_{8}$	10	0	0	0	0	0	0	0	0	0	0	0	0	0	0	0	0	0	0	0	0	0	0	0	0	0	0	0	0	0	0	0	0	0	0	0	0	0	0	0	0	0	0	0
$s_{9}$	100	0	0	0	0	0	0	0	0	0	0	0	0	0	0	0	0	0	0	0	0	0	0	0	0	0	0	0	0	0	0	0	0	0	0	0	0	0	0	0	0	0	0	0
$s_{10}$	100	0	0	0	0	0	0	0	0	0	0	0	0	0	0	0	0	0	0	0	0	0	0	0	0	0	0	0	0	0	0	0	0	0	0	0	0	0	0	0	0	0	0	0
$s_{11}$	100	0	0	1	0	0	0	0	0	0	0	0	0	0	0	0	0	0	0	0	0	0	0	0	0	0	0	0	0	0	0	1	0	0	0	0	0	1	0	0	0	0	0	0
$s_{12}$	100	0	0	0	1	0	0	0	0	0	0	0	0	0	0	0	0	0	0	0	0	0	0	0	0	0	0	0	0	0	0	1	0	0	0	0	0	1	0	0	0	0	0	0
$s_{13}$	10	0	0	0	1	0	0	0	0	0	0	0	0	0	0	0	0	0	0	0	0	0	0	0	0	0	0	0	0	0	0	1	0	0	0	0	0	1	0	0	0	0	0	0
$s_{14}$	100	0	0	0	0	0	0	0	0	0	0	0	0	0	0	0	0	0	0	0	0	0	0	0	0	0	0	0	0	0	0	0	0	0	0	0	0	0	0	0	0	0	0	0
$s_{15}$	100	0	0	0	0	0	0	0	0	0	0	0	0	0	0	0	0	0	0	0	0	0	0	0	0	0	0	0	0	0	0	0	0	0	0	0	0	0	0	0	0	0	0	0
$s_{16}$	100	0	0	0	0	1	0	0	0	0	0	0	0	0	0	0	0	0	0	0	0	0	0	0	0	0	0	0	0	0	0	1	0	0	0	0	0	1	0	0	0	0	0	0
$s_{17}$	100	0	0	0	0	0	1	0	0	0	0	0	0	0	0	0	0	0	0	0	0	0	0	0	0	0	0	0	0	0	0	1	0	0	0	0	0	1	0	0	0	0	0	0
$s_{18}$	10	0	0	0	0	0	1	0	0	0	0	0	0	0	0	0	0	0	0	0	0	0	0	0	0	0	0	0	0	0	0	1	0	0	0	0	0	1	0	0	0	0	0	0
$s_{19}$	10	0	0	0	0	0	0	1	0	0	0	0	0	0	0	0	0	0	0	0	0	0	0	0	0	0	0	0	0	0	0	1	0	0	0	0	0	1	0	0	0	0	0	0
$s_{20}$	1	0	0	0	0	0	0	1	1	0	0	0	0	0	0	0	0	0	0	0	0	0	0	0	0	0	0	0	0	0	0	1	0	0	0	0	0	1	0	0	0	0	0	0
$s_{21}$	100	0	0	0	0	0	0	0	1	0	0	0	0	0	0	0	0	0	0	0	0	0	0	0	0	0	0	0	0	0	0	1	0	0	0	0	0	1	0	0	0	0	0	0
$s_{22}$	10	0	0	0	0	0	0	0	0	0	0	0	0	0	0	0	0	0	0	0	0	0	0	0	0	0	0	0	0	0	0	0	0	0	0	0	0	0	0	0	0	0	0	0
$s_{23}$	100	0	0	0	0	0	0	0	0	1	0	0	0	0	0	0	0	0	0	0	0	0	0	0	0	0	0	0	0	0	0	1	0	0	0	0	0	1	0	0	0	0	0	0
$s_{24}$	100	0	0	0	0	0	0	0	0	0	1	0	0	0	0	0	0	0	0	0	0	0	0	0	0	0	0	0	0	0	0	0	0	0	0	0	0	0	1	0	0	0	0	0
$s_{25}$	10	0	0	0	0	0	0	0	0	0	0	1	0	0	0	0	0	0	0	0	0	0	0	0	0	0	0	0	0	0	0	0	0	0	0	0	0	0	0	0	0	0	0	0
$s_{26}$	10	0	0	0	0	0	0	0	0	0	0	0	1	0	0	0	0	0	0	0	0	0	0	0	0	0	0	0	0	0	0	1	0	0	0	0	0	1	0	0	0	0	0	0
$s_{27}$	10	0	0	0	0	0	0	0	0	0	0	0	1	0	0	0	0	0	0	0	0	0	0	0	0	0	0	0	0	0	0	1	0	0	0	0	0	1	0	0	0	0	0	0
$s_{28}$	10	0	0	0	0	0	0	0	0	0	0	0	0	1	0	0	0	0	0	0	0	0	0	0	0	0	0	0	0	0	0	1	0	0	0	0	0	1	0	0	0	0	0	0
$s_{29}$	1	0	0	0	0	0	0	0	0	0	0	0	0	1	1	0	0	0	0	0	0	0	0	0	0	0	0	0	0	0	0	1	0	0	0	0	0	1	0	0	0	0	0	0
$s_{30}$	10	0	0	0	0	0	0	0	0	0	0	0	0	0	0	1	0	0	0	0	0	0	0	0	0	0	0	0	0	0	0	1	0	0	0	0	0	1	0	0	0	0	0	0
$s_{31}$	1	0	0	0	0	0	0	0	0	0	0	0	0	0	0	1	1	0	0	0	0	0	0	0	0	0	0	0	0	0	0	1	0	0	0	0	0	1	0	0	0	0	0	0
$s_{32}$	100	0	0	0	0	0	0	0	0	0	0	0	0	0	0	0	0	1	0	0	0	0	0	0	0	0	0	0	0	0	0	1	0	0	0	0	0	1	0	0	0	0	0	0
$s_{33}$	100	0	0	0	0	0	0	0	0	0	0	0	0	0	0	0	0	0	1	0	0	0	0	0	0	0	0	0	0	0	0	0	0	0	0	0	0	0	1	0	0	0	0	0
$s_{34}$	1	0	0	0	0	0	0	0	0	0	0	0	0	0	0	0	0	0	0	1	0	0	0	0	0	0	0	0	0	0	0	0	0	0	0	0	0	0	0	0	0	1	0	0
$s_{35}$	10	0	0	0	0	0	0	0	0	0	0	0	0	0	0	0	0	1	1	1	1	0	0	0	0	0	0	0	0	0	0	1	0	0	1	0	0	1	0	0	0	1	0	0
$s_{36}$	100	0	0	0	0	0	0	0	0	0	0	0	0	0	0	0	0	0	0	0	0	1	0	0	0	0	0	0	0	0	0	1	0	0	0	0	0	1	0	0	0	0	1	0
$s_{37}$	1	0	0	0	0	0	0	0	0	0	0	0	0	0	0	0	0	0	0	0	0	1	1	0	0	0	0	0	0	0	0	1	0	0	0	0	0	1	0	0	0	0	1	0
$s_{38}$	1	0	0	0	0	0	0	0	0	0	0	0	0	0	0	0	0	0	0	0	0	1	1	1	0	0	0	0	0	0	0	1	0	0	1	0	0	1	0	0	0	0	1	0
$s_{39}$	10	0	0	0	0	0	0	0	0	0	0	0	0	0	0	0	0	0	0	0	0	0	0	0	1	1	0	0	0	0	0	0	0	0	0	0	0	0	0	0	0	0	0	0
$s_{40}$	10	0	0	0	0	0	0	0	0	0	0	0	0	0	0	0	0	0	0	0	0	0	0	0	0	0	1	0	0	0	0	0	0	0	0	0	0	0	0	0	0	0	1	0
$s_{41}$	10	0	0	0	0	0	0	1	0	1	0	0	1	0	0	0	0	0	0	0	0	1	1	1	0	0	0	1	0	0	0	1	0	0	1	0	0	1	0	0	0	0	1	0
$s_{42}$	10	0	0	0	0	0	0	1	0	1	0	0	1	0	0	0	0	0	0	0	0	0	0	0	0	0	0	1	0	0	0	1	0	0	0	0	0	1	0	0	0	0	0	0
$s_{43}$	10	0	0	0	0	0	0	0	0	0	0	0	0	0	0	0	0	0	0	0	0	0	0	0	0	0	0	0	0	0	0	0	0	0	0	0	0	0	0	0	0	0	0	0
$s_{44}$	1	0	0	0	0	0	0	1	0	1	0	0	1	0	0	0	0	0	0	0	0	0	0	0	0	0	0	0	0	0	0	1	0	0	0	0	0	1	0	0	0	0	0	0
$s_{45}$	1	0	0	0	0	0	0	0	0	0	0	0	0	0	0	0	0	0	0	0	1	0	0	1	0	0	0	0	0	1	0	1	0	0	0	0	0	0	0	0	0	0	1	0
$s_{46}$	1	0	0	0	0	0	0	0	0	0	0	0	0	0	0	0	0	0	0	0	0	0	0	0	0	0	0	0	1	0	0	1	0	0	0	0	0	0	0	0	0	0	0	0
$s_{47}$	10	0	0	0	0	0	0	0	0	0	0	0	0	0	0	0	0	0	0	0	0	0	0	0	0	0	0	0	0	0	1	0	0	0	1	1	0	0	0	0	0	0	0	0
$s_{48}$	1	0	0	0	0	0	0	0	0	0	0	0	0	0	0	0	0	0	0	0	0	0	0	0	0	0	0	0	1	1	1	1	0	0	1	1	0	1	0	0	0	0	0	1
$s_{49}$	10	0	0	0	0	0	0	1	0	1	0	0	1	0	0	0	0	0	0	0	0	0	0	0	0	0	0	1	0	0	0	1	1	0	0	0	0	1	0	0	0	0	0	0
$s_{50}$	10	0	0	0	0	0	0	0	0	0	0	0	0	0	0	0	0	0	0	0	0	0	0	0	0	0	0	0	0	0	0	0	0	1	0	0	1	1	0	0	0	0	0	0
$s_{51}$	10	0	0	0	0	0	0	0	0	0	0	0	0	0	0	0	0	0	0	0	0	0	0	0	0	0	0	0	0	0	0	0	0	0	1	1	0	0	0	0	0	0	0	0
$s_{52}$	1	0	0	0	0	0	0	0	0	0	0	0	0	0	0	0	0	0	0	0	0	0	0	0	0	0	0	0	0	0	0	0	0	0	0	0	0	0	0	0	0	0	0	0
$s_{53}$	10	0	0	0	0	0	0	0	0	0	0	0	0	0	0	0	0	0	0	0	0	0	0	0	0	0	0	0	0	0	0	0	0	0	0	0	0	0	0	0	0	0	0	0
$s_{54}$	100	0	0	0	0	0	0	0	0	0	0	0	0	0	0	0	0	0	0	0	0	0	0	0	0	0	0	0	0	0	0	1	0	0	0	0	0	1	0	1	0	0	0	0
$s_{55}$	100	0	0	0	0	0	0	0	0	0	0	0	0	0	0	0	0	0	0	0	0	0	0	0	0	0	0	0	0	0	0	1	0	0	0	0	0	1	0	0	1	0	0	0
$s_{56}$	1	0	0	0	0	0	0	0	0	0	0	0	0	0	0	0	0	0	0	1	0	0	0	0	0	0	0	0	0	0	0	0	0	0	0	0	0	0	0	0	0	0	0	0
$s_{57}$	1	0	0	0	0	0	0	0	0	0	0	0	0	0	0	0	0	0	0	0	0	0	0	0	0	0	0	0	0	0	0	1	0	0	0	0	0	1	0	0	0	0	0	1

## Appendix B. Time-Constrained Diagnostic Trees of the Measurement Device

The three nominal-prior trees use the D/P/C in Appendix A and include only tests completed within normalized Time 36. Blue and red links denote responses 0 and 1. Terminal labels show the stored MAP decision; the associated candidate sets are listed with each tree. Ambiguous terminal sets contribute through that MAP decision.
